# Development of novel pyrimidine nucleoside analogs as potential anticancer agents: Synthesis, characterization, and *In-vitro* evaluation against pancreatic cancer

**DOI:** 10.1016/j.ejps.2024.106754

**Published:** 2024-03-29

**Authors:** Esther Frimpong, Raviteja Bulusu, Joy Okoro, Andriana Inkoom, Nkafu Ndemazie, Sherise Rogers, Xue Zhu, Bo Han, Edward Agyare

**Affiliations:** a College of Pharmacy and Pharmaceutical Sciences, Florida A&M University, Tallahassee, FL, United States; b Department of Medicine, Division of Hematology and Oncology, University of Florida College of Medicine, Gainesville, FL, United States; c Department of Surgery, Keck School of Medicine, University of Southern California, Los Angeles, CA, United States; d Department of Internal Medicine, Richmond University Medical Center, Staten Island, NY, United States

**Keywords:** Pyrimidine nucleosides, Pancreatic cancer, Cytotoxicity, Synthesis, 5-FU, Characterization

## Abstract

The present study proposed modification of 5-FU by conjugation with an acyl chloride and a 5-membered heterocyclic ring to improve its *in-vitro* cytotoxicity and metabolic stability. XYZ-I-71 and XYZ-I-73 were synthesized by introducing a tetrahydrofuran ring on 5-fluorocytosine (a precursor of 5-FU) and conjugation with octanoyl chloride and lauroyl chloride, respectively. The structure of the synthesized compounds was validated using NMR and micro-elemental analysis. The antiproliferative activity of the analogs was determined against MiaPaCa-2, PANC-1, and BxPC-3 pancreatic cancer cells. The analog’s stability in human liver microsomes was quantified by HPLC. We found that the XYZ-I-73 (IC_50_ 3.6 ± 0.4 μM) analog was most effective against MiaPaCa-2 cells compared to XYZ-I-71(IC_50_ 12.3 ± 1.7 μM), GemHCl (IC_50_ 24.2 ± 1.3 μM), Irinotecan (IC_50_ 10.1 ± 1.5 μM**)** and 5-FU (IC_50_ 13.2 ± 1.1 μM**)**. The antiproliferative effects of this analog in Miapaca-2 cells is evident based on it having a 7-fold,3-fold, and 4-fold increased cytotoxic effect over Gem-HCl, Irinotecan, and 5-FU, respectively. On the other hand, XYZ-I-71 exhibited a 2-fold increased cytotoxic effect over Gem-HCl but a comparable cytotoxic effect to 5-FU and Irinotecan in MiaPaCa-2 cells. A similar trend of higher XYZ-I-73 inhibition was observed in PANC-1 and BxPC-3 cultures. For 48-h MiaPaCa-2 cell migration studies, XYZ-I-73 (5 μM) significantly reduced migration (# of migrated cells, 168 ± 2.9), followed by XYZ-I-71(315±2.1), Gem-HCl (762±3.1) and 5-FU (710 ± 3.2). PARP absorbance studies demonstrated significant inhibition of PARP expression of XYZ-I-73 treated cells compared to 5-FU, GemHCl, and XYZ-I-71. Further, BAX and p53 expressions were significantly increased in cells treated with XYZ-I-73 compared to 5-FU, GemHCl, and XYZ-I-71. *In-vitro*, metabolic stability studies showed that 80 ± 5.9% of XYZ-I-71 and XYZ-I-73 remained intact after 2 h exposure in liver microsomal solution compared to 5-FU. The XYZ-I-73 analog demonstrated a remarkable cytotoxic effect and improved *in-vitro* metabolic stability over the selected standard drugs and may have potential anticancer activity against pancreatic cancer.

## 1. Introduction

Pancreatic cancer is a highly aggressive solid tumor characterized by a poor prognosis and a low survival rate (Michl and Gress, 2013; Wang et al., 2017). In the United States, about 3 % of all cancers and 7 % of all cancer deaths are due to pancreatic cancer (Grossberg et al., 2020). The American Cancer Society estimated an incidence of 64,050 cases for 2023, with 50,550 deaths, making it the third most deadly cancer in the United States (Siegel et al., 2023). African Americans have the highest incidence and pancreatic cancer-related mortality among all racial groups in the United States (Khawja et al., 2015; Tavakkoli et al., 2020). The higher mortality rates among African Americans have been partly attributed to genetic polymorphism affecting the metabolism of chemotherapeutic agents and some social determinants of health (Khawja et al., 2015). Tumors of the pancreas can emanate from either the endocrine or exocrine pancreatic component, with the most common and aggressive forms emanating from the latter (Torphy et al., 2020). Risk factors for pancreatic cancer can be classified as modifiable and non-modifiable (Midha et al., 2016). The modifiable risk factors include smoking, obesity, alcohol, *Helicobacter pylori*, and dietary factors. In contrast, the non-modifiable risk factors include age, sex, ethnicity, blood group, diabetes, family history, and genetic association (McGuigan et al., 2018).

Early diagnosis of pancreatic cancer poses a significant challenge to overall survival, as many patients are asymptomatic and majorly present at the advanced or metastasized stage of the disease (Ashok et al., 2022). About 90 % of patients are diagnosed at the advanced stage, where surgical resection is almost ineffective (Torphy et al., 2020). Chemotherapy has become the primary standardized treatment. However, this comes with high toxicities and drug resistance that significantly contributes to a less than 12.5 % survival rate within five years of diagnosis associated with the disease (Adiseshaiah et al., 2016; Siegel et al., 2023). Furthermore, the formation of desmoplasia around pancreatic cancer cells poses a significant challenge in managing the disease, as the matrix’s components have been implicated in drug resistance (Mei et al., 2016).

Despite advancements in oncology treatment globally, there are limited treatment options for pancreatic cancer. Gemcitabine (Gem), a known anticancer agent, is the standard treatment for pancreatic cancer (Immordino et al., 2004; Wood et al., 2022), however, rapid metabolism (short half-life) has limited its use (de Sousa Cavalcante and Monteiro, 2014). Further, Gem undergoes rapid deamination catalyzed by cytidine deaminase to its inactive (2,2-difluorodeoxyuridine) derivative, resulting in its short half-life (Pili et al., 2010).

5-FU, a pyrimidine nucleoside, is a known anticancer drug for treating a broad range of tumors, including pancreatic, colorectal, and breast cancers (Vodenkova et al., 2020). Its mechanism of action is through the inhibition of thymidylate synthase and the incorporation of its metabolites into deoxyribonucleic acid (DNA) and ribonucleic acid (RNA) after it is transported into cancer cells via the uracil transport system (Ghafouri-Fard et al., 2021). 5-FU exerts its anticancer activity through its active metabolites: Fluorouridine triphosphate (FUTP), Fluorodeoxyuridine triphosphate (FdUTP), and Fluorodeoxyuridine monophosphate FdUMP (Miura et al., 2010). FUTP disrupts RNA synthesis, while FdUTP and FdUMP cause DNA damage; both processes ultimately lead to apoptosis (Miura et al., 2010). Some researchers have previously questioned the benefits of 5-FU in managing pancreatic cancer (Wang et al., 2014). This was prompted by the results of a randomized study involving 126 subjects to determine the efficacy of GemHCl and 5-FU in patients newly diagnosed with advanced pancreatic cancer (Burris 3rd et al., 1997). The trial showed that GemHCl had a slightly better overall survival over 5-FU, with a median survival of 5.65 months vs. 1.4 months; *p* = 0.0025, and a 1-year survival rate of 18 % for GemHCl patients and 2 % for 5-FU (Burris 3rd et al., 1997). Subsequent research, however, has proven the benefits of 5-FU, especially as a combination therapy in managing pancreatic cancer (Wang et al., 2014).

Folfirinox, a combination therapy comprising (5-FU, leucovorin, irinotecan, and oxaliplatin) has been shown to be beneficial for patients with metastasized or advanced pancreatic cancer (Conroy et al., 2013). A study conducted to determine the efficacy of Folfirinox and GemHCl in patients with metastasized pancreatic cancer established the benefits of Folfirinox over GemHCl (Conroy et al., 2011). Folfirinox gave a better response (31.6% vs. 9.4 % *p <* 0.001), progression-free survival (6.4 vs. 3.3 months; *p <* 0.001), and overall survival (11.1 months vs. 6.8 months; *p <* 0.001) over GemHCl (Conroy et al., 2011). However, Folfirinox is limited to use in a few patients, and its benefit in locally advanced pancreatic adenocarcinomas still needs to be established (Conroy et al., 2013).

Due to the shortcomings associated with the standard therapy, there has been a growing interest in applying modified existing chemotherapeutic agents in cancer research (Hidalgo et al., 2014; Jin et al., 2010). Thymoquinone analogs attached with gallate and fluorogallate pharmacophores and combined with or without GemHCl showed higher cytotoxic activity than the unmodified drug in MiaPaCa-2 and BxPC-3 pancreatic cancer cell lines (Yusufi et al., 2013).

The drawbacks associated with GemHCl and Folfirinox have warranted a desperate need for new and efficient anticancer agents in managing pancreatic cancer. The proven superior cytotoxic activity of analogs of chemotherapeutic agents led to this study. In the present study, two novel 5-FU analogs were developed via chemical modification of the parent molecule 5-FU by attaching a tetrahydrofuran ring at the C-1 position and an amide at the C-4 position. This study focused on the details of the drug design, synthesis, and *in-vitro* cytotoxicity studies against the MiaPaCa-2, PANC-1, and BxPC-3 pancreatic cancer cell lines.

## 2. Materials and methods

### 2.1. Materials

All chemicals, 5-FU, and analytical-grade reagents were purchased from Sigma-Aldrich (St. Louis, Missouri, USA). Gem-HCl and Cytosine were purchased from AK Scientific (Union City, CA). Pancreatic cancer MiaPaCa-2 cells (K-Ras (G12C)) were bought from American Type Culture Collection (ATCC) (Manassas, VA).

### 2.2. Synthesis of 5-FU analogs

#### 2.2.1. Synthesis of XYZ-I-71

**Scheme 1.**
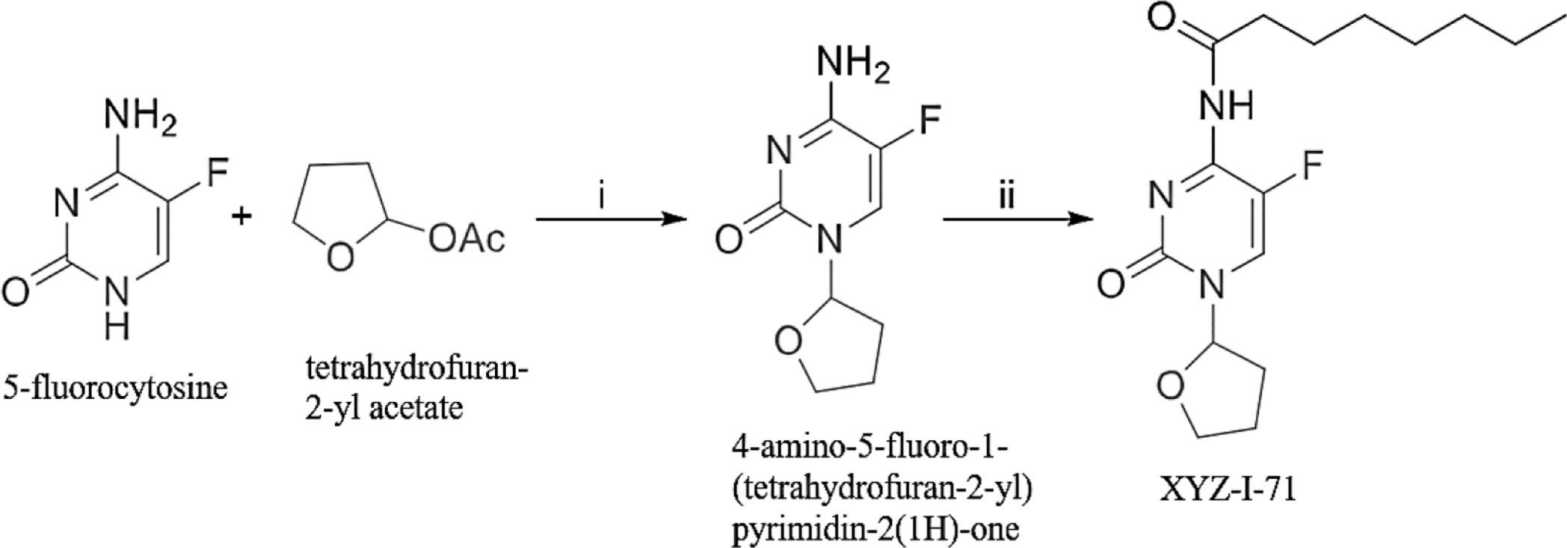
Synthesis of XYZ-I-71.

XYZ-I-71 was synthesized according to a method described by Zasada et al., with some modifications as depicted in Scheme 1 (Zasada et al., 2017). A mixture of 5-fluorocytosine (2.0 g, 15.50 mmol), tetrahydrofuran-2-yl acetate (4.2 g, 32.31 mmol), 1,8-Diazabicyclo [5.4.0] undec‑7-ene (DBU) (5.0 g, 32.89 mmol) in pyridine (20 mL) was heated with stirring at 95 °C for 48 h. in a sealed flask. After cooling to room temperature, the reaction was diluted with EtOAc (400 mL) and washed with water (300×2 mL). The aqueous solution was collected and concentrated in *vacuo* to dryness. The residue was dried under vacuum for 48 h. and followed by crystallization (MeOH/EtOAc/Hexane) to give 4-amino-5-fluoro-1-(tetrahydrofuran-2-yl) pyrimidin-2(1H)-one, 2.25 g, in a yield of 73 %.

##### NMR data of 4-amino-5-fluoro-1-(tetrahydrofuran-2-yl) pyrimidin-2 (1H)-one (intermediate compound)

^1^H NMR (DMSO‑d^6^, 300 MHz) δ 7.71 (1H, d, *J* = 7.2 Hz), 7.66 (1H, brs), 7.42 (1H, brs), 5.81 – 5.84 (1H, m), 4.20 (1H, dd, *J* = 5.7, 12.9 Hz), 3.76 (1H, dd, *J* = 7.2, 12.9 Hz), 2.10 – 2.21 (1H, m), 1.80 – 1.94 (3H, m).

^13^C NMR (DMSO‑d^6^, 151 MHz) δ 157.9 (d, *J* = 13.2 Hz), 153.8, 136.5 (d, *J* = 240.6 Hz), 125.7 (d, *J* = 31.2 Hz), 87.1, 69.6, 32.5, 23.9.

4-amino-5-fluoro-1,-(tetrahydrofuran-2-yl) pyrimidin-2(1H)-one (1.0 g, 5.02 mmol) in pyridine (10 mL) was added while stirring to a solution of octanoyl chloride (1 g, 6.15 mmol) in CH_2_Cl_2_ (5 mL) dropwise at 0 °C in 30 min. The solution was stirred at room temperature for 12 h. The reaction was diluted with EtOAc (300 mL) and then washed with saturated NaHCO_3_ (100 mL), and brine (100 mL). The organic layer was dried with Na_2_SO_4_, filtered, and concentrated in *vacuo* to dryness. The residue was purified on silica gel on an Isolera chomatograph with gradient eluant (Hexane/EtOAc). After crystallization from EtOAc/Hexane, N-(5-fluoro-2-oxo-1-(tetrahydrofuran-2-yl)−1,2-dihydropyrimidin-4-yl) octanamide was obtained, 1.22 g, in a yield of 74 %.

Log P: 2.27; Rf (Hexane/EtOAc, 1/3): 0.30; MP (melting point):

119–120 °C; Purity was greater than 99.6 %

NMR data for N-(5-fluoro-2-oxo-1-(tetrahydrofuran-2-yl)−1,2-dihydropyrimidin-4-yl) octanamide (XYZ-I-71)

^1^H NMR (CDCl3, 300 MHz) δ 7.80 (1H, brs), 7.67 (1H, d, *J* = 6.0 Hz), 5.94 (1H, dd, *J* = 1.5, 5.1 Hz), 4.24 (1H, dt, *J* = 3.9, 8.1 Hz), 4.04 (1H, q, *J* = 7.8 Hz), 2.97 (2H, brs), 2.43–2.55 (1H, m), 2.08 – 2.18 (1H, m), 2.00 – 2.05 (1H, m), 1.78 – 1.89 (1H, m), 1.63 – 1.73 (2H, m), 1.22 – 1.38 (8H, m), 0.87 (3H, t, *J* = 6.9 Hz).

^13^C NMR (CDCl3, 151 MHz) δ 174.6, 152.9 (d, *J* = 12.4 Hz), 152.2, 136.8 (d, *J* = 245.8 Hz), 127.6 (d, *J* = 31.4 Hz), 88.8, 70.5, 38.0, 33.0, 31.6, 29.0, 28.9, 24.6, 23.4, 22.5,14.0.

*Calcd for C*_*16*_*H*_*24*_*FN*_*3*_*O*_*3*_: C 59.06, H 7.43, N 12.91; *Found*: C 58.95, H 7.34, N 12.82. Molecular weight: 325.38, Log P: 2.27. Purity was greater than 99.6 % based on the calculation of elemental analysis.

#### 2.2.2. Synthesis of XYZ-I-73

**Scheme 2.**
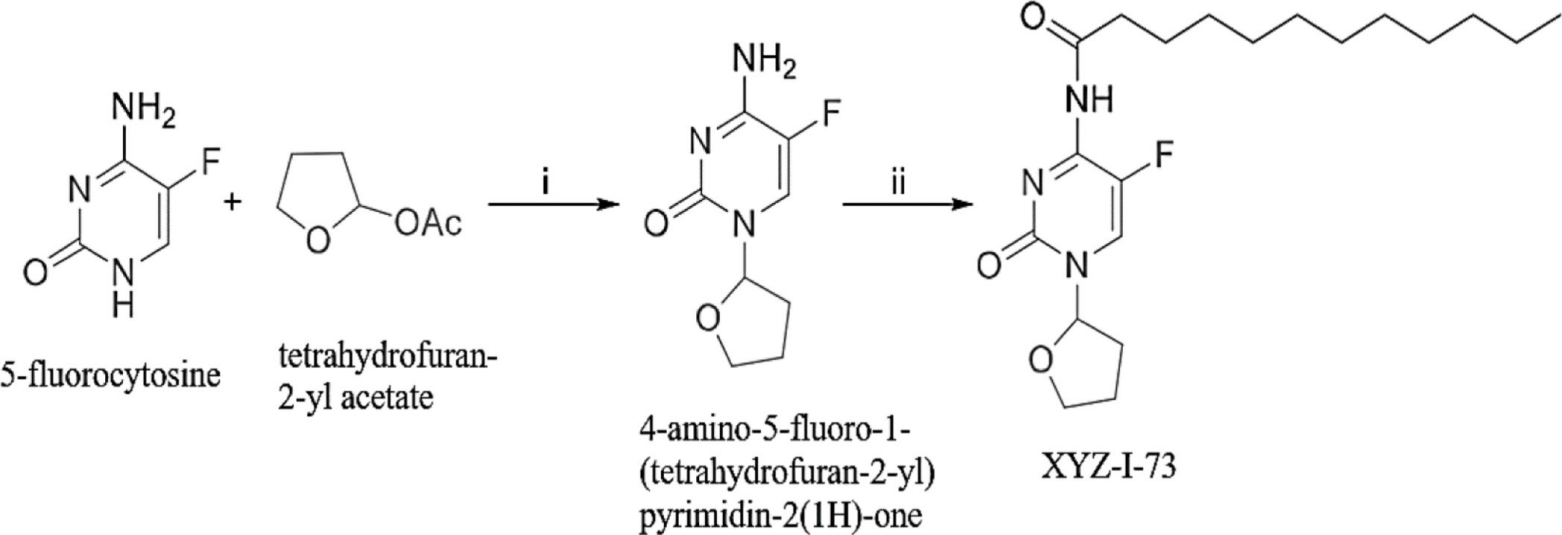
Synthesis of XYZ-I-73.

XYZ-I-73 was synthesized according to a method described by Zasada et al., with some modifications as depicted in Scheme 2 (Zasada et al., 2017). A mixture of 5-fluorocytosine (2.0 g, 15.50 mmol), tetrahydrofuran-2-yl acetate (4.2 g, 32.31 mmol), 1,8-Diazabicyclo [5.4.0] undec‑7-ene (DBU) (5.0 g, 32.89 mmol) in pyridine (20 mL) was heated with stirring at 95 °C for 48 h. in a sealed flask. After cooling to room temperature, the reaction was diluted with EtOAc (400 mL) and washed with water (300×2 mL). The aqueous solution was collected and concentrated in *vacuo* to dryness. The residue was dried under vacuum for 48 h. and followed by crystallization (MeOH/EtOAc/Hexane) to give 4-amino-5-fluoro-1-(tetrahydrofuran-2-yl) pyrimidin-2(1H)-one, 2.25 g, in a yield of 73 %.

##### NMR data of 4-amino-5-fluoro-1-(tetrahydrofuran-2-yl) pyrimidin-2 (_1_H)-one (intermediate compound)

^1^H NMR (DMSO‑d^6^, 300 MHz) δ 7.71 (1H, d, *J* = 7.2 Hz), 7.66 (1H, brs), 7.42 (1H, brs), 5.81 – 5.84 (1H, m), 4.20 (1H, dd, *J* = 5.7, 12.9 Hz), 3.76 (1H, dd, *J* = 7.2, 12.9 Hz), 2.10 – 2.21 (1H, m), 1.80 – 1.94 (3H, m).

^13^C NMR (DMSO‑d^6^, 151 MHz) δ 157.9 (d, *J* = 13.2 Hz), 153.8, 136.5 (d, *J* = 240.6 Hz), 125.7 (d, *J* = 31.2 Hz), 87.1, 69.6, 32.5, 23.9.

4-amino-5-fluoro-1-(tetrahydrofuran-2-yl) pyrimidin-2(1H)-one (1.0 g, 5.02 mmol) in pyridine (10 mL) was added with stirring to a solution of lauroyl chloride (1.31 g, 6.03 mmol) in CH_2_Cl_2_ (5 mL) dropwise at 0 °C in 30 min. The solution was stirred at room temperature for 12 h. The reaction was diluted with EtOAc (300 mL) and followed by washing with sat. NaHCO_3_ (100 mL), brine (100 mL). The organic layer was dried with Na_2_SO_4_, filtered, and concentrated in *vacuo* to dryness. The residue was purified on silica gel on an Isolera chomatograph with gradient eluant (Hexane/EtOAc). After crystallization from EtOAc/Hexane, yielded N-(5-fluoro-2-oxo-1-(tetrahydrofuran-2-yl)−1,2-dihydropyrimidin-4-yl) dodecanamide, 1.34 g, in a yield of 70 %.

Log P: 3.94; *Rf* (Hexane/EtOAc, 1/3): 0.32.; MP (melting point): 107–108 °C, Purity was greater than 99.6 %.

NMR data for N-(5-fluoro-2-oxo-1-(tetrahydrofuran-2-yl)−1,2-dihydropyrimidin-4-yl) dodecanamide (XYZ-I-73)

^1^H NMR (CDCl_3_, 300 MHz) δ 7.73 (1H, brs), 7.67 (1H, d, *J* = 5.4 Hz), 5.94 (1H, dd, *J* = 1.5, 5.1 Hz), 4.24 (1H, dt, *J* = 3.9, 7.5 Hz), 4.02 (1H, q, *J* = 8.7 Hz), 2.99 (2H, brs), 2.42 – 2.56 (1H, m), 2.10 – 2.19 (1H, m), 2.00 – 2.07 (1H, m), 1.79 – 1.89 (1H, m), 1.66 − 1.73 (2H, m), 1.18 – 1.38 (16H, m), 0.87 (3H, t, *J* = 6.9 Hz).

^13^C NMR (CDCl_3_, 151 MHz) δ 174.19, 152.91 (d, *J* = 13.8 Hz), 152.46, 156.73 (d, *J* = 244.9 Hz), 127.65 (d, *J* = 31.6 Hz), 88.76, 70.54, 37.98, 33.01, 31.84, 29.56, 29.44, 29.41, 29.27, 29.04, 24.46, 23.41, 22.61, 14.04.

*Calcd for C*_*20*_*H*_*32*_*FN*_*3*_*O*_*3*_: C 62.97, H 8.46, N 11.01; *Found*: C 62.77, H 8.51, N 10.83. Molecular weight: 381.49, Log P: 3.94. Purity was greater than 99.6 % based on the calculation of elemental analysis.

2.3. *In-vitro* cell viability studies testing the efficacy of analogs XYZ-I-71 and XYZ-I-73 against MiaPaCa-2, PANC-1, and BxPC-3 pancreatic cancer cells

Prior to viability studies, Dulbecco’s Modified Eagle Medium (DMEM with high glucose and l- glutamine), was supplemented with 10 % fetal bovine serum (FBS) and 1 % penicillin-streptomycin (PenStrep) (Vande Voorde et al., 2019). Briefly, MiaPaCa-2 and PANC-1 cells were seeded at a density of 6 × 10^3^ per well in 96-well plates in triplicates for each drug concentration level and incubated at 5 % CO_2_ and a temperature of 37 °C (Vande Voorde et al., 2019). BxPC-3 cells in Roswell Park Memorial Institute (RPMI 1640 with HEPES) supplemented with 10 % FBS and 1 % PenStrep were cultured similarly. At 70 − 75 % confluence, the cells were treated with compounds XYZ-I-71, XYZ-I-73,5-FU, Gem-HCl, and Irinotecan. Varying concentrations of XYZ-I-71, XYZ-I-73, and Irinotecan were prepared from its stock solution and serially diluted with the growth medium. For 5-FU and Gem-HCl, a stock solution was prepared with phosphate-buffered saline (PBS) and serially diluted with growth medium to prepare varied concentrations: thus 3, 6, 12, 25, and 100 μM. The cells were treated with 200 μL of each drug concentration in triplicates and incubated for 48 h. At termination, 20 μL of 0.05 % resazurin sodium salt (Alamar blue^®^) was added and incubated at optimum conditions (5 % CO_2_, 37 °C) for 4 h (Gradiz et al., 2016). Fluorometric analysis was determined at an excitation wavelength of 560/580 nm and emission wavelength of 590/610 nm, and the percent viable cells per concentration was calculated.

### 2.3. Cell migration assay

Cell migration assay was conducted to determine the effect of 5-FU, Gem-HCl, XYZ- I-71, and XYZ-I-73 on MiaPaCa-2 cell motility. Ibidi cell culture inserts were used to generate two confluent monolayers of cells separated by a “wound” for this assay. The cells were seeded in 24-well plates at a cell density of 2.5× 10^5^ for 24 h at 37 °C. At 75 % confluency, cells formed adherent monolayers on either side of the tissue culture insert. Prior to treatment, the cells were serum-starved by replacing complete media with base media (DMEM) and then further incubated for 24 h. After the cells had been serum starved, the insert was gently removed to generate a gap or “wound” between the two confluent layers of cells. The monolayers were washed with experimental media, and afterward, cells were exposed to varying concentrations of 5-FU, GemHCl, XYZ-I-71, and XYZ-I-73 (1, 2, 5, and 10 μM). Images of the cells invading the wound were captured with an Olympus DP70 Camera. Migrated cells within the scratch were quantified, and the migration distance was measured using NIH ImageJ software (Ntantie et al., 2017).

### 2.4. Western blot (PARP apoptosis) study

Western blot was performed as previously described (Kalvala et al., 2023; Mondal and Bennett, 2016). Briefly, MiaPaCa-2 cells were seeded in 6 well plate-containing DMEM supplemented with 10 % FBS at a cell density of 2.5× 10^5^ cells per well. On reaching optimum confluency, cells were exposed to drug treatment at IC_50_/2 and IC_50_ concentrations of XYZ-I-71, XYZ-I-73, 5-FU and GemHCl for 12 h. Further, cells were washed in PBS, and whole cell lysate was prepared using RIPA buffer containing protease and phosphatase inhibitor (1:100) (Sigma Aldrich’s. Louis, MO, USA). This was followed by 30 min incubation on ice, and subsequently, the supernatant was collected after centrifuging at 12,000 rpm for 20 min. The protein estimation of supernatant was performed using BCA protein assay. The Sodium Dodecyl Sulfate–Polyacrylamide Gel Electrophoresis (SDS-PAGE) loading sample was prepared by heating at 98 °C for 10 min. A sample containing 40 μg equivalent proteins was allowed to run on SDS-PAGE and then transferred onto Polyvinylidene fluoride membrane (PVDF), followed by blocking in 5 % BSA in Tris-Buffered Saline Tween 20(TBST). The membrane was incubated with primary antibodies, PARP (Poly (ADP-ribose) polymerase) (1:1000), p53 (1:1000), BAX (Bcl-2-associated X protein) (1:1000), and β-actin (1:1000) (CST, USA), prepared in TBST at 40 °C overnight on a shaker. After washing, membranes were incubated with Horseradish peroxidase (HP) - conjugated secondary antibodies (Cell signaling technology, USA) (1:20,000), and chemiluminescence was visualized using a Fusion-FX chemiluminescence imager (Vilber Lourmat, Germany). The relative band densities were quantified using software (Image J 1.36; Wayne Rasband, National Institute of Health, MD, USA).

### 2.5. PARP absorbance study

PARP enzyme activity on MiaPaca-2 cells was estimated using the higher throughput (HT) 96 test Colorimetric PARP/Apoptosis Assay Kit with Histone-Coated Strip Wells (Trevigen, Gaitherburg, MD, USA) according to the manufacturer’s instructions and previous studies (Navarro et al., 2017). Miapaca-2 cells were seeded in 96 well plate at a cell density of 1.5 × 10^4^ cells per well containing DMEM supplemented with 10 % FBS. The cells were treated with XYZ-I-71, XYZ-I-73, 5-FU and GemHCl at their respective IC_50_ concentrations and incubated for 2, 4, and 6 h. Triplicates of each treatment were maintained. Aspiration and washing with PBS were done with extreme care to avoid the loss of apoptotic cells. Cell lysates were prepared using a cell extraction buffer, and the protein concentration was determined using BCA protein assay. Protein concentration was adjusted to 200 ng protein / 25 μL. Thereafter, histone-coated plates were activated for 30 min using PARP buffer. The next step was the ribosylation reaction, in which 25 μL of cell lysate in triplicates and PARP substrate cocktail was added into each well and incubated for 30 min. After incubation, the strip wells were washed four times with PBS and 0.1% Triton X-100. 50 μL of diluted anti-PARP monoclonal antibody was added and incubated for 1.5 h at room temperature, followed by washing four times and incubation for another 1.5 h with anti-mouse IgG-HP conjugate. Subsequently, the strips were washed four times, added 50 μL of TACS-Sapphire to each well, and incubated for 5–10 min in the dark. The ribosylation reaction step was terminated by adding 50 μL of 0.2 M HCl, and thereafter, absorbance was read at 450 nm.

### 2.6. Human liver microsome (HLM) stability of XYZ-I-71 and XYZ-I-73

Human liver microsome stability of XYZ-I-71 and XYZ-I-73 was determined according to the manufacturer’s protocol (ThermoFisher Scientific). XYZ-I-71 and XYZ-I-73 (concentration 1000 ppm) were prepared in a suitable solvent. The microsomes were thawed slowly on ice, and the concentration was adjusted to 20 mg/mL.183 μL of 100 mM phosphate buffer, 2 uL 1000 ppm Test article, and 5 uL of 20 mg/mL microsomes were added to a tube. The microsomes, buffer, and test article were pre-incubated in a water bath at 37 °C for 5 min. The incubated samples were shaken for a specified time at 150 rpm. The reaction was initiated by adding 10 μL 20 mM NADPH, and the resultant mixture was incubated up to 240 min at 37 °C with gentle agitation. The process included the incubation times (0, 5,15, 30, 60,120 and 240 min). The blank samples were the same composition as the test samples without any biologically active compounds. The process was terminated with 200 μL of ice-cold methanol. The samples were vortexed and centrifuged at approximately 3000 rpm for five minutes. The supernatant was withdrawn from the protein pellet. After that, samples were immediately prepared and analyzed using HPLC. All samples were prepared in triplicate.

### 2.7. Statistical analysis

For the statistical analysis, results are presented as mean ± SEM. Data was analyzed for significance by one-way ANOVA followed by Tukey’s Multiple Comparison Test using GraphPad Prism 9 Software, and IC_50_ values were determined.

## 3. Results

### 3.1. Synthesis and characterization of XYZ-I-71 and XYZ-I-73

We successfully synthesized XYZ-I-71(Scheme 1) and XYZ-I-73 (Scheme 2) in good yields and characterized their structures using ^1^H and ^13^C NMR (Supplementary Figs. S_1_ and S_2_), micro elemental analysis, HPLC and mass spectrometry. Characteristic XYZ-I-71 ^1^H NMR peak was observed at 7.80 ppm, and ^13^C NMR peaks were observed at 174.6 ppm and 152.9 ppm, representing the amide bond formed from the conjugation of octanoyl chloride to the 5-fluorocutosine cyclic structure. Characteristic XYZ-I-73 ^1^H NMR peak was observed at 7.73 ppm, and ^13^C NMR peaks were observed at 174.19 ppm and 152.91 ppm, representing the amide bond formed from the conjugation of lauroyl chloride to the 5-fluorocytosine cyclic structure. These peaks confirmed the successful conjugation of octanoyl and lauroyl chloride on 5-fluorocytosine.

Reagents and conditions for the synthesis of XYZ-I-71 i) DBU, Pyridine, 95 °C, 48 h; ii) Acyl chloride (Octanoyl chloride), pyridine, rt.

Reagents and conditions for synthesis of XYZ-I-73: i) DBU, Pyridine, 95 °C,48 h; ii) Acyl chloride (Lauroyl chloride), pyridine, rt.

#### 3.1.1. Micro-elemental analysis

The elemental analysis of XYZ-I-71 and XYX-I-73 is presented in Tables 1 and 2, respectively. For XYZ-I-71, the analysis indicated that there was 58.95 % carbon, 7.34 % hydrogen, and 12.82 % nitrogen compared with the theoretical values of 59.06 % carbon, 7.43 % hydrogen, and 12.91 %. For XYZ-I-73, it indicated that there is 62.77 % of carbon, 8.51 % of hydrogen, and 10.83 % nitrogen compared with the theoretical values of 62.97 % carbon, 8.46 % of hydrogen, and 11.01 %. The purity of both compounds was greater than 99.6 % based on the elemental analysis (Supplementary Figs. S_3_ and S_4_).

#### 3.1.2. HPLC and mass spectrometry

The HPLC analysis showed the purity for XYZ-I-71 and XYX-I-73 was greater than 99.6 %, indicative of pure compounds synthesized. The retention time was 3.62 min for XYZ-I-71 and 5.77 min for XYZ-I-73, as shown in Supplementary Figs. S_5_ and S_6_. For the liquid chromatography-mass spectrometry (LC-MS) analysis, the highest intensities were at a mass-to-charge ratio (*m/z* ratio) of 325.80 and 381.88, corresponding to the molecular weights of XYZ-I-71 and XYX-I-73 respectively (Supplementary Figs. S_7_ and S_8_).

3.2. *In-vitro* cell viability studies testing the efficacy of compounds XYZ-I-71 and XYZ-I-73 against MiaPaCa-2, PANC-1, and BxPC-3 pancreatic cancer cells

**Fig. 1.**
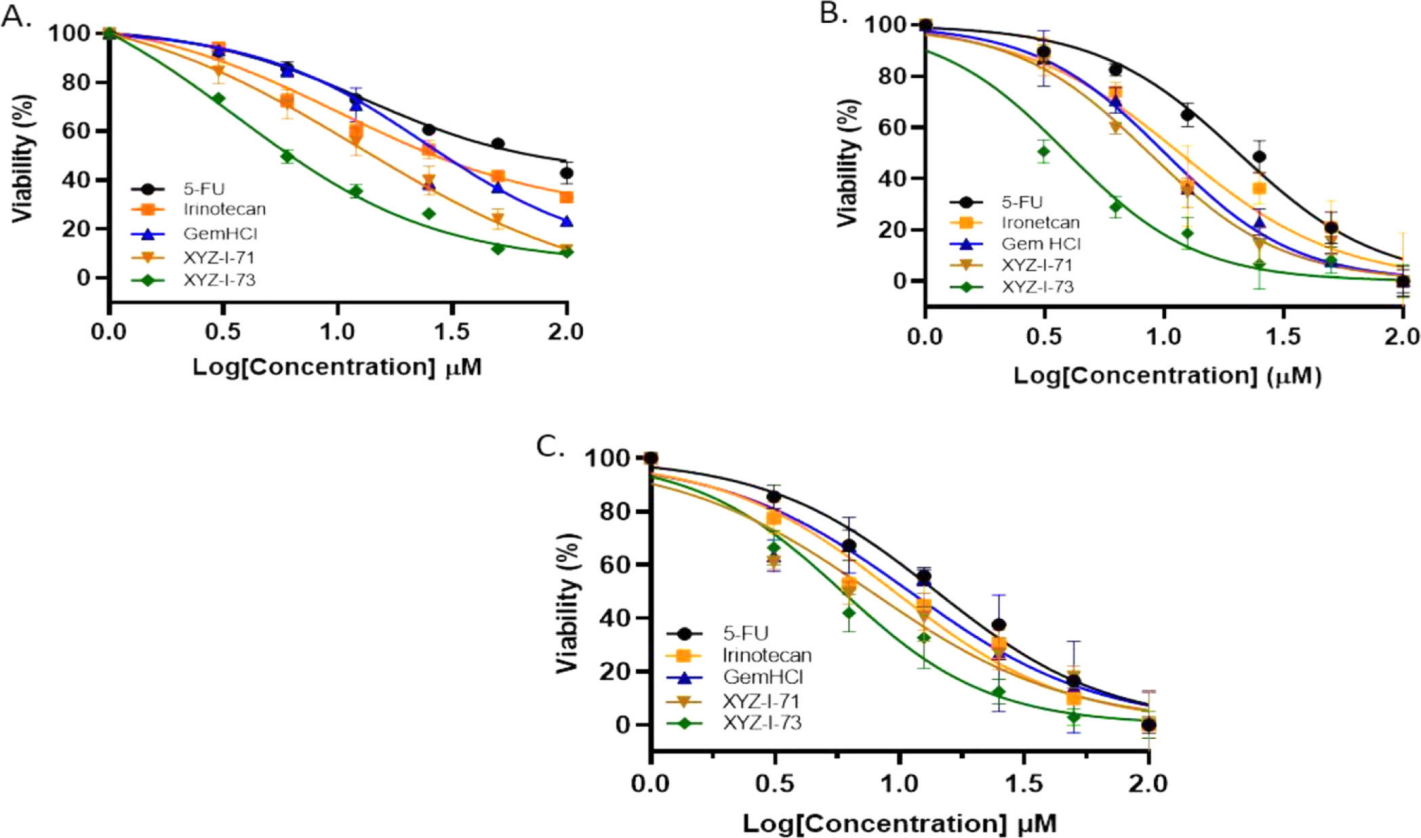
Cytotoxic activity of XYZ-1–71, XYZ-I-73, 5-FU, GemHCl, and Irinotecan on MiaPaCa-2, PANC-1, and BxPC-3 after 48 h incubation.

The analogs (XYZ-I-71 and XYZ-I-73) were shown to have a good cytotoxic effect against the MiaPaCa-2 cancer cells. The IC_50_ values of the two newly synthesized 5-FU analogs were found to be 12.3 ± 1.7 μM (XYZ-1–71) and 3.6 ± 0.4 μM (XYZ-1–73). The IC_50_ values of the analogs were compared with the standard drugs 5-FU, Gem-HCl, and Irinotecan (13.2 ± 1.1 μM, 24.2 ± 1.3 μM, and 10.1 ± 1.5 μM), respectively, which are depicted in Fig.1a and Table 3. These results show that XYZ-I-73 had about a 7-fold increased cytotoxic effect over Gem-HCl (IC_50_ 24.2 ± 1.3 μM), a 3-fold increased cytotoxic effect over Irinotecan (IC_50_ 10.1 ± 1.5 μM**)**, and a 4-fold increased cytotoxic effect over the parent compound, 5-FU (IC_50_ 13.2 ± 1.1 μM). XYZ-I-71 showed a higher cytotoxic activity over Gem-HCl but comparable activity to 5-FU and Irinotecan. Moreover, the analog XYZ-I-73 had a higher cytotoxic effect than the XYZ-71, as shown in Fig. 1A. Cytotoxic effect of XYZ-I-73 treated PANC-1 culture (IC_50_ 3.92±0.5) was remarkably higher than GemHCl (IC_50_ 10.07±0.9), 5-FU (IC_50_ 20.43±1.2) and Irinotecan (IC_50_ 11.63±1.1). XYZ-I-71(IC_50_ 8.65±0.9), on the other hand, had a higher cytotoxic effect than 5-FU and Irinotecan but comparable cytotoxicity with GemHCl as shown in Fig. 1B. A similar trend of higher XYZ-I-73 (IC_50_ 5.88±0.7) inhibition in BxPC-3 culture was found compared to 5-FU (IC_50_ 14.02±1.1), GemHCl (IC_50_ 10.95±0.9), and Irinotecan (IC_50_ 9.51±1.0) as shown in Fig. 1C. and Table 3. XYZ-I-71(IC_50_ 7.66±0.8) showed higher cytotoxicity than 5-FU and GemHCl but comparable cytotoxicity to Irinotecan (Fig. 1C). Overall, XYZ-I-73 proved to have superior cytotoxicity in Mia-PaCa-2, PANC-1, and BxPC-3 cells than GemHCl, 5-FU and Irinotecan treated cancer cells.

Cytotoxic studies on (a) MiaPaCa-2 cells treated with XYZ-1–71, XYZ-I-73, 5-FU, GemHCl, and Irinotecan, (b) PANC-1 cells treated with XYZ-1–71, XYZ-I-73, 5-FU, GemHCl, and Irinotecan, (c) BxPC-3 cells treated with XYZ-1–71, XYZ-I-73, 5-FU, GemHCl, and Irinotecan. IC_50_ values for XYZ-I-73 were significantly lower than those of GemHCl, 5-FU, and Irinotecan, implying a higher cytotoxic activity. IC_50_ value for XYZ-I-71 was lower than GemHCl but comparable to Irinotecan and 5-FU in MiaPaCa-2 cells. Data represent mean ± SEM, *n* = 3. (***p < 0.001 (XYZ-I-73 (IC_50_) vs Gem HCl (IC_50_), 5-FU (IC_50_)), (***p < 0.001 (XYZ-I-71 (IC_50_) vs. Gem HCl (IC_50_)).

### 3.2. In-vitro cell migration studies testing the efficacy of compounds XYZ-I-71 and XYZ-I-73 against MiaPaCa-2 pancreatic cancer cells

**Fig. 2.**
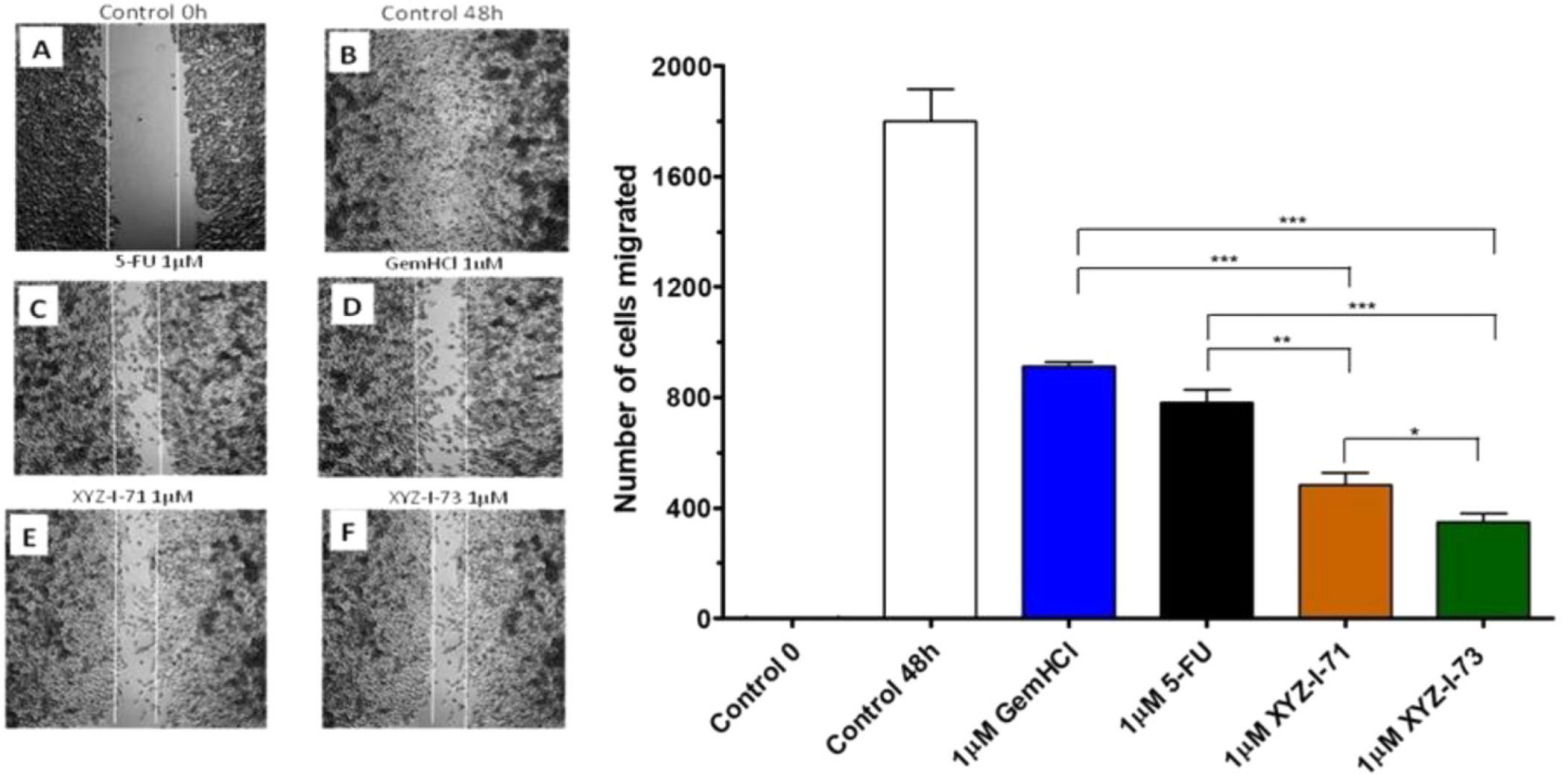
Cell migration study of XYZ-1–71, XYZ-I-73, 5-FU, and GemHCl on MiaPaCa-2 cells at 1 μM after 48 h incubation.

**Fig. 3.**
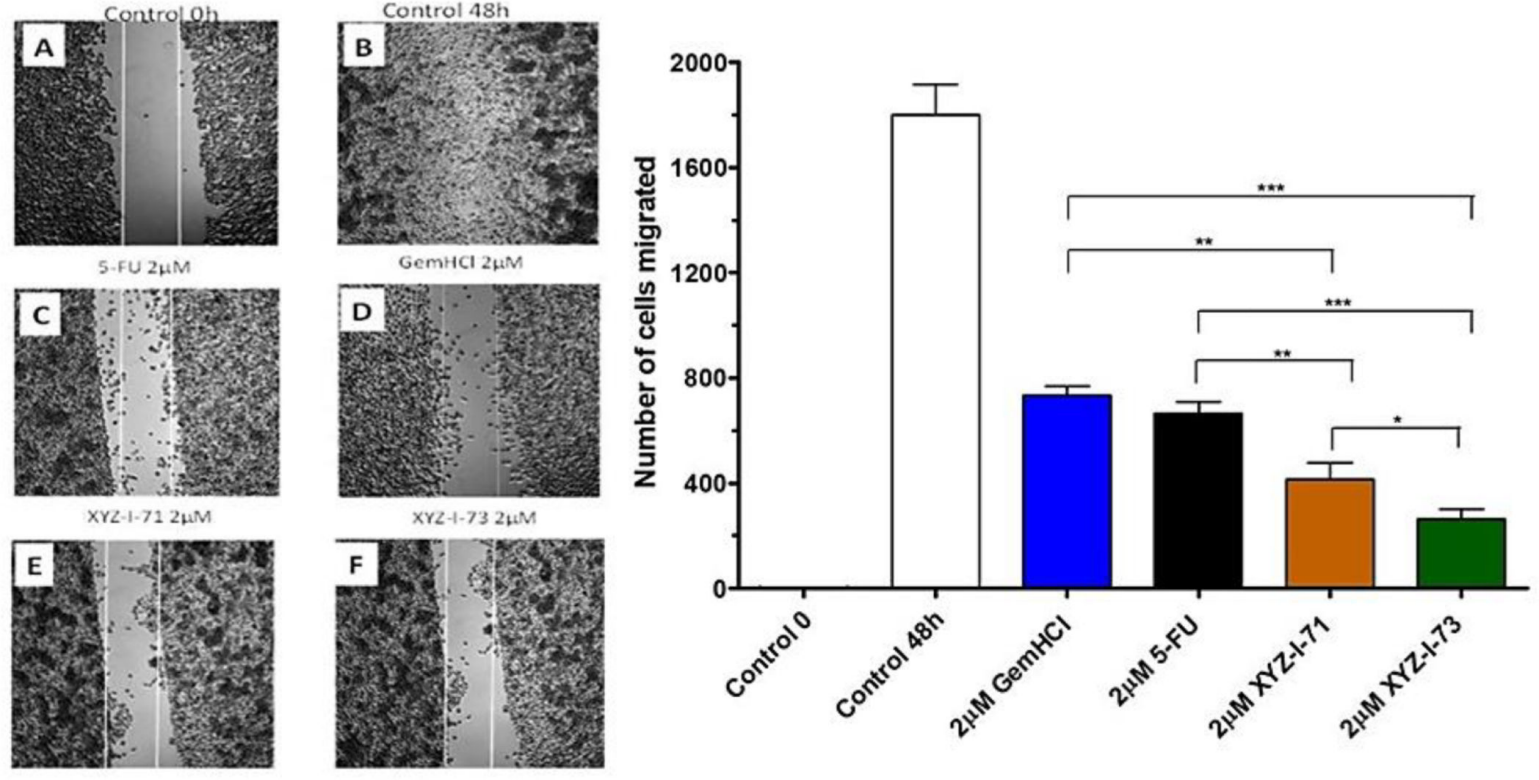
Cell migration study of XYZ-1–71, XYZ-I-73, 5-FU, and GemHCl on MiaPaCa-2 cells at 2 μM after 48 h incubation.

**Fig. 4.**
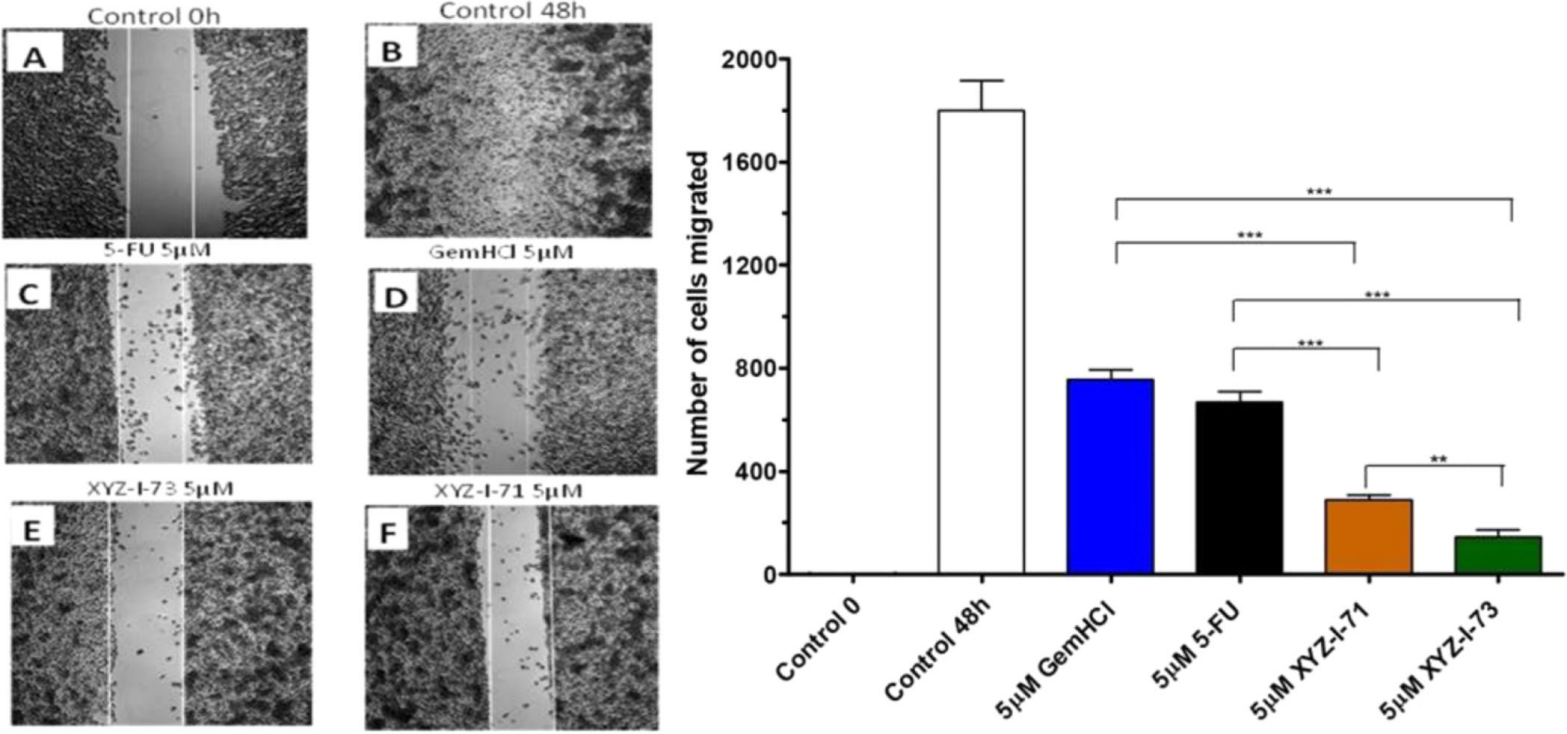
Cell migration study of XYZ-1–71, XYZ-I-73, 5-FU, and GemHCl on MiaPaCa-2 cells at 5 μM after 48 h incubation.

**Fig. 5.**
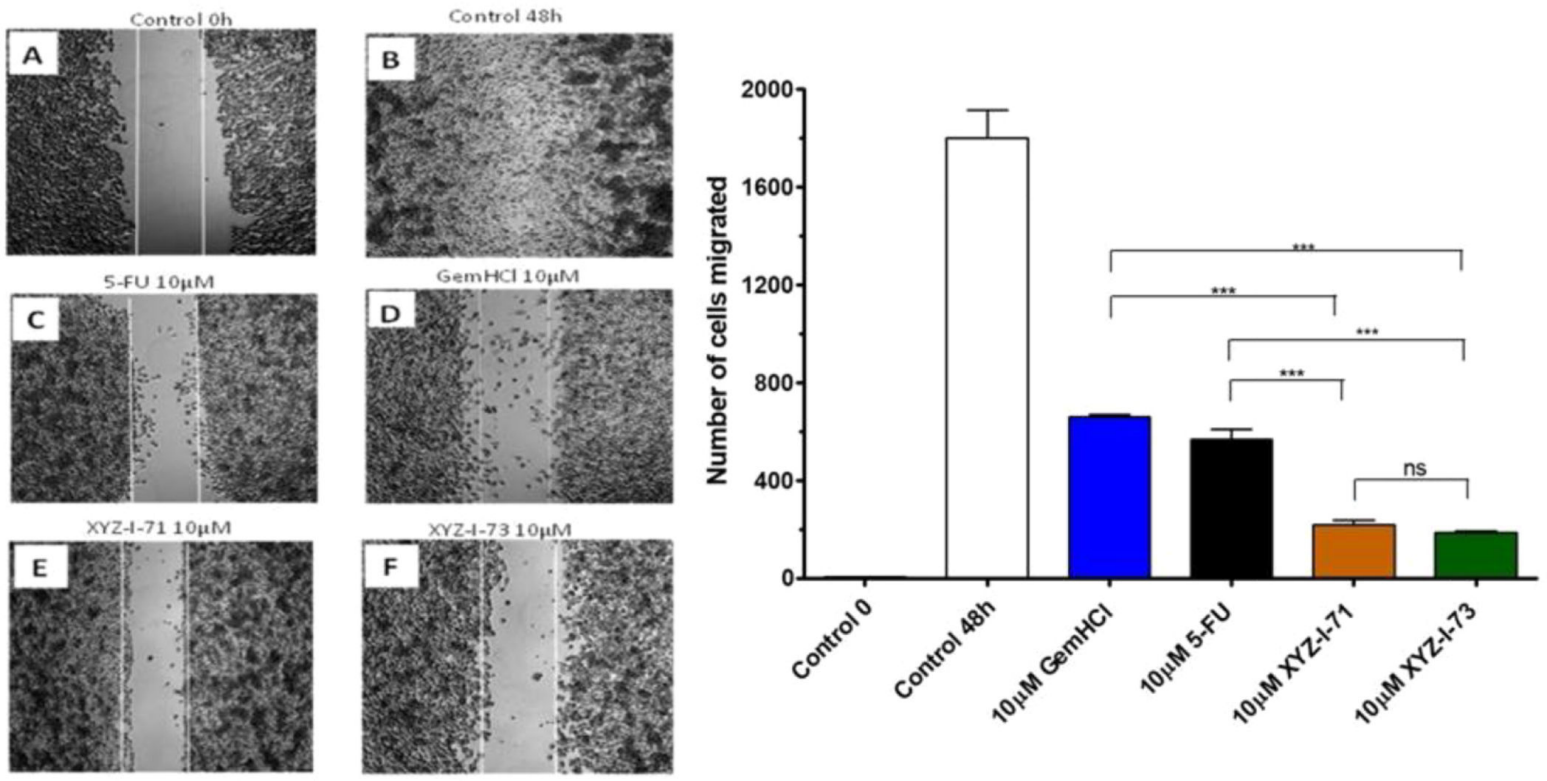
Cell migration study of XYZ-1–71, XYZ-I-73, 5-FU, and GemHCl on MiaPaCa-2 cells at 10 μM after 48 h incubation.

This study was performed on MiaPaCa-2 cells treated with 5-FU, Gem-HCl, and the novel analogs XYZ-I-71 and XYZ-I-73 at different concentrations ranging from 1 to 10 μM, as shown in Figs. 2–5. From the data, concentrations of 1, 2, 5, and 10 μM of XYZ-I-73 and XYZ-1–71 significantly inhibited the cell movement toward the wound compared to standard drugs after 48 h. Over the same incubation time, the cells in the control group (48 h) migrated farther, achieving a complete or near closure of the wounds. As depicted in Fig. 2 and Table 4, XYZ-I-71 and XYZ-I-73 treated cells at 1 μM concentration significantly reduced cell motility towards the wound area with (485±3.5) and (376±2.9) cells migrated respectively compared to Gem-HCl (878±2.1) and 5-FU (790 ±4.3). A similar trend in cell migration toward the wound was observed at concentrations of 2 μM (Fig. 3), 5 μM (Fig. 4), and 10 μM (Fig. 5) of the analogs and the standard drugs. The results are expressed as the means (± SEM, *n* = 3) relative to the control.

XYZ-I-73 significantly inhibited the migration of MiaPaca-2 cells compared to the 5-FU, Gem-HCl, Irinotecan and XYZ-I-71. A= Control at 0 h; B=Control at 48 h; C = 5-FU at 48 h; *D*=Gem-HCl at 48 h; *E*= XYZ-I-71 at 48 h; *F*=XYZ-I-73 at 48 h; *G*=No. of migrated cells for control and treatments after 48 h Data represent mean ± SEM, *n* = 3. (***p < 0.001 (XYZ-I-73 _(IC50)_ vs Gem HCl _(IC50)_, 5-FU _(IC50)_).

XYZ-I-73 significantly inhibited the migration of MiaPaca-2 cells compared to the 5-FU, Gem-HCl, Irinotecan and XYZ-I-71. A= Control at 0 h; B=Control at 48 h; C = 5-FU at 48 h; *D*=Gem-HCl at 48 h; *E*= XYZ-I-71 at 48 h; *F*=XYZ-I-73 at 48 h; *G*=No. of migrated cells for control and treatments after 48 h Data represent mean ± SEM, *n* = 3. (***p < 0.001 (XYZ-I-73 _(IC50)_ vs Gem HCl _(IC50)_, 5-FU _(IC50)_).

XYZ-I-73 significantly inhibited the migration of MiaPaca-2 cells compared to the 5-FU, Gem-HCl, Irinotecan and XYZ-I-71. A= Control at 0 h; B=Control at 48 h; C = 5-FU at 48 h; *D*=Gem-HCl at 48 h; *E*= XYZ-I-71 at 48 h; *F*=XYZ-I-73 at 48 h; *G*=No. of migrated cells for control and treatments after 48 h Data represent mean ± SEM, *n* = 3. (***p < 0.001 (XYZ-I-73 _(IC50)_ vs Gem HCl _(IC50)_, 5-FU _(IC50)_).

XYZ-I-73 significantly inhibited the migration of MiaPaCa-2 cells compared to the 5-FU, Gem-HCl, Irinotecan and XYZ-I-71. A= Control at 0 h; B=Control at 48 h; C = 5-FU at 48 h; *D*=Gem-HCl at 48 h; *E*= XYZ-I-71 at 48 h; *F*=XYZ-I-73 at 48 h; *G*=No. of migrated cells for control and treatments after 48 h Data represent mean ± SEM, *n* = 3. (***p < 0.001 (XYZ-I-73 _(IC50)_ vs Gem HCl _(IC50)_, 5-FU _(IC50)_).

### 3.3. Western blot analysis

**Fig. 6.**
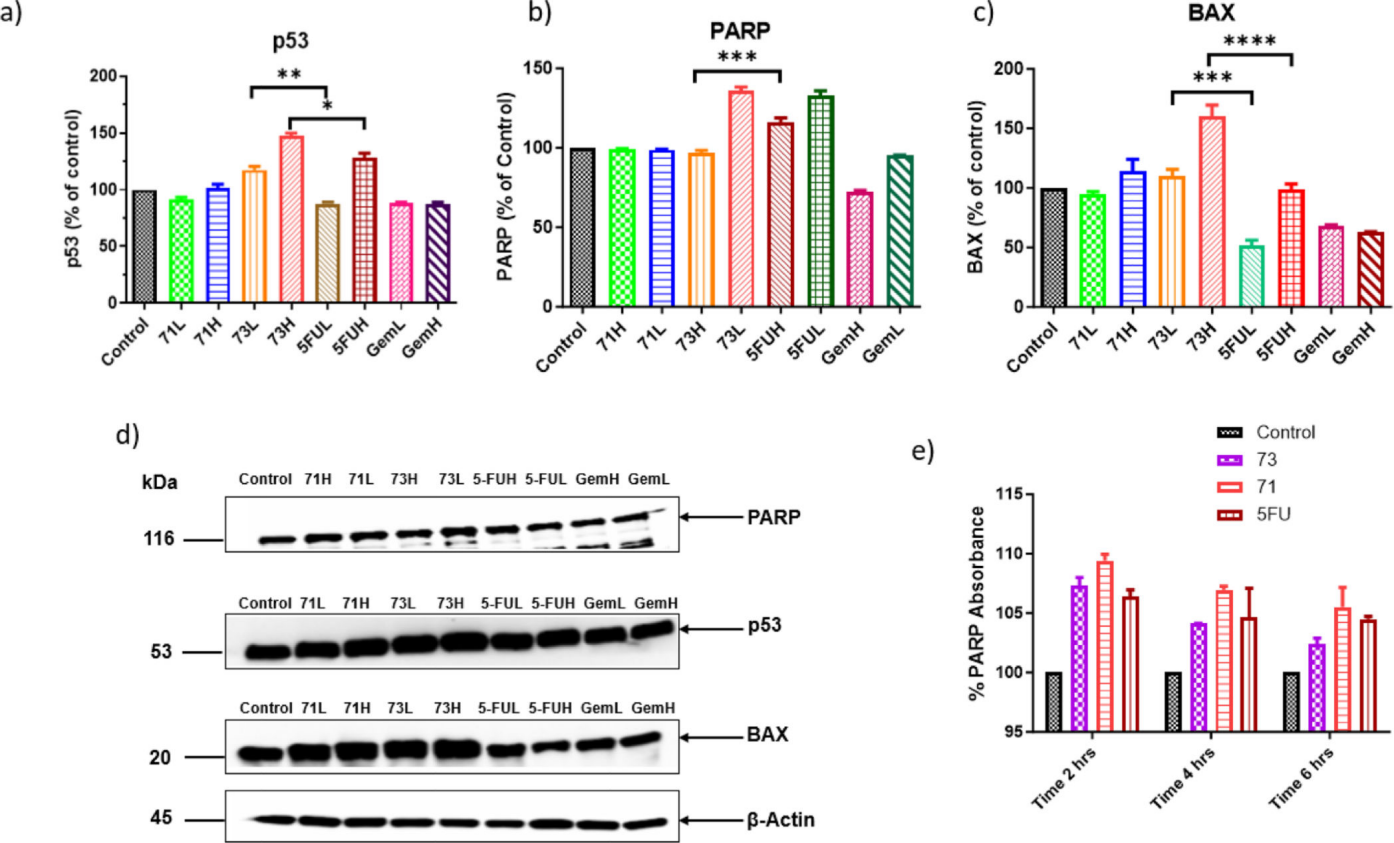
Protein expression in MiaPaCa-2 cells after 12 h treatment with IC_50_/2 & IC_50_ concentrations of XYZ-1–71, XYZ-I-73, 5-FU, and GemHCl.

The level of tumor suppression protein p53 was increased with the increase in the concentrations of novel analogs, and a significant increase in the expression of p53 was observed with the compound XYZ-I-73 when compared to 5-FU and the control (Fig. 6a). The level of the PARP protein expression was higher in the lower concentrations of novel analogs. A decrease in PARP levels was observed as the concentration of drugs increased, suggesting that the compound could induce apoptosis in cancer cells (Fig. 6b). Similar to the tumor suppression protein p53, a significant increase was observed in the levels of BAX apoptotic protein when treated with IC_50_ concentrations of XYZ-I-73, when compared to other treatments and control (Fig. 6c). Fig. 6d shows a blot of the proteins expressed after the treatment with the novel analogs (XYZ-I-71 and XYZ-I-73) and 5-FU.

IC_50_/2 & IC_50_ concentrations for respective drugs are indicated as L & H, respectively. For protein band expression, the blots were cut prior to hybridization with primary antibodies during the blotting. a) Quantitative expressions of tumor suppression protein p53 b) Quantitative expressions of PARP protein c) Quantitative expression of apoptotic protein BAX d) Expressions of proteins upon treatment; Full-length blots are presented in Supplementary Fig. S_9_ and e) PARP absorbance% when incubated for 2, 4 and 6 h with IC_50_ concentration.

### 3.4. PARP absorbance study

The absorbance readings at 450 nm showed a reduction in PARP activity as the incubation period increased, as shown in Fig. 8e. XYZ-I- 71 and XYZ-I- 73 were able to reduce the PARP protein concentration, and a clear inhibiting trend was observed. The compound XYZ-I- 73 showed a higher reduction in PARP protein concentration when incubated for 6 h compared with 5-FU and Gem-HCl (Fig. 6e).

### 3.5. In-vitro metabolic stability of XYZ-I-71 and XYZ-I-73 using human liver microsomes

**Fig. 7.**
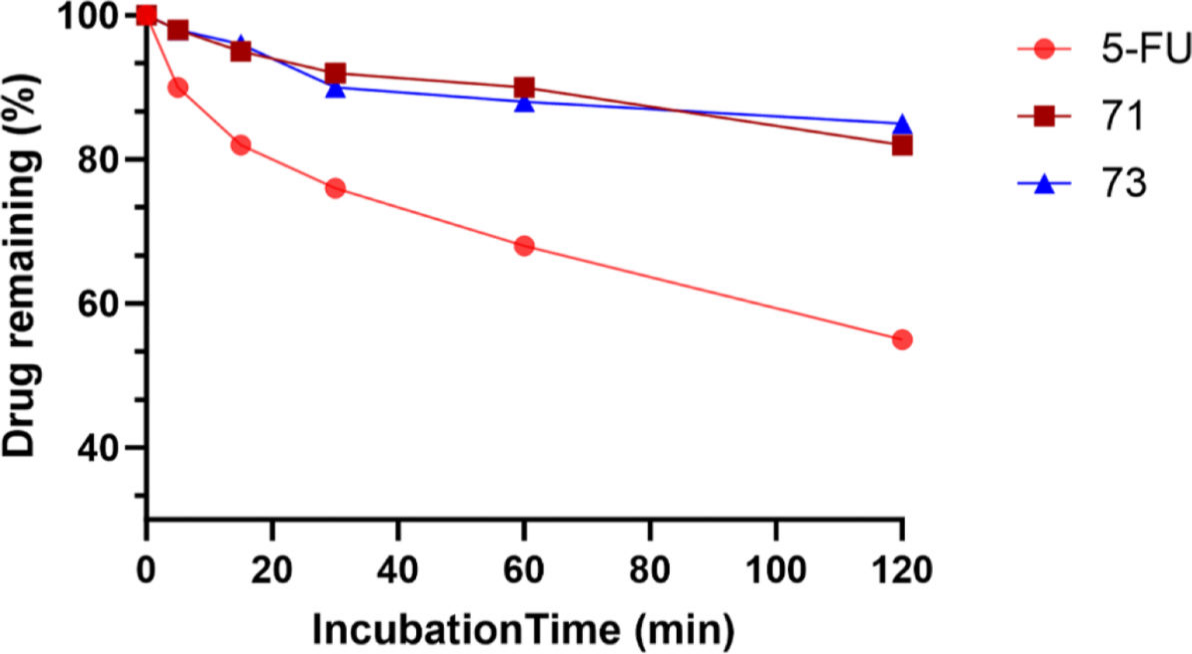
*In-vitro* metabolic stability of XYZ-I-71, XYZ-1–73 and 5-FU in human liver microsomes after exposure for 2 h.

In-vitro metabolism of XYZ-I-71 and XYZ-I-73 in human liver microsomes was investigated using HPLC, and the findings are shown in Fig. 7. Percent unchanged or intact XYZ-I-71 and XYZ-I-73 were plotted against different time points. The HPLC analysis of the percent XYZ-I-71 and XYZ-I-73 remaining was greater than 80 ± 5.9 % after 2 h. However, the metabolic stability of 5-FU using human liver microsomes revealed less than 55±4.3 % of intact 5-FU remaining after 2 h. This implies that XYZ-I-71 and XYZ-I-73 may have better metabolic stability compared with 5-FU.

Percentage of XYZ-I-71, XYZ-1–73, and 5-FU remaining shown after exposure to human liver microsomes over 2 h. Data represent mean ± SEM, *n* = 3.

## 4. Discussion

5-FU, a known anticancer agent, has been used for almost seven decades in treating colorectal, pancreatic, breast, head, and cervical cancers (Zhang et al., 2008). It is a prodrug and undergoes a series of phosphorylation reactions leading to its activation and subsequent anticancer activity. It has been found to be rapidly metabolized when administered intravenously, resulting in a short half-life of about 10–25 min (Wigmore et al., 2010). Further, almost 90 % of administered dose is metabolized to inactive dihydroflurouracil by dihydropyrimidine dehydrogenase in the liver and by peripheral blood mononuclear cells, intestinal mucosa, and kidneys, limiting its efficacy (Lollo et al., 2019). This necessitates administering the anticancer drug at a high dose for an extended period to give the desired therapeutic effect (Gusella et al., 2006).

To address these limitations associated with 5-FU, we developed and synthesized novel 5-FU analogs by conjugating a tetrahydrofuran ring at the C-1 position and an amide group at the C-4 position of 5-fluorocytosine to produce XYZ-I- 71 and XYZ-I-73 (Schemes 1 and 2). The rationale for synthesizing the analogs was to improve metabolic stability, prolong systemic circulation, and enhance the cytotoxic effect of 5-FU. These conjugations, especially with long-chain hydrocarbons, decrease the rapid metabolism/inactivation of the parent compound, ultimately leading to increased systemic stability and enhanced cytotoxic activity (Rautio et al., 2018). According to Immordino and colleagues, the advantage of selecting long-chain hydrocarbons such as XYZ-I-73 over short-chain hydrocarbons, for example, XYZ-I-71, is the ease with which high amount can be loaded into liposomal delivery system (Immordino et al., 2004).

Prior to starting the *in-vitro* studies, the structure and purity of XYZ-I-71 and XYZ-I-73 were confirmed using NMR, HPLC, mass spectrometry, and elemental analysis. NMR was used to characterize these analogs, i.e., study the chemical analogs’ structures to confirm the amide bond’s presence in the newly synthesized compounds (Supplementary Figs. S_3_ and S_4_). The elemental analysis also matched the synthesized compounds’ calculated carbon, hydrogen, and nitrogen presence, confirming the compounds’ purity as shown in Tables 1 and 2. The purity of XYZ-I-71 and XYZ-I-73 was 99.6 % for both compounds, suggesting that the synthesized compounds were significantly devoid of unwanted material.

The *in-vitro* cell viability (2D cell model) data obtained after treating MiaPaCa-2, PANC-1, and BxPC-3 cells with the novel 5-FU analogs suggested that these compounds could be possible therapeutic agents in treating pancreatic cancer (Fig. 1). The IC_50_ value of XYZ-I-73 in MiaPaCa-2, PANC-1, and BxPC-3 cancer cells was significantly lower than that of Gem-HCl, Irinotecan, and 5-FU, suggesting a higher cytotoxic effect. The antiproliferative effects of this analog in Miapaca-2 cells is evident based on it having a 7-fold,3-fold, and 4-fold increased cytotoxic effect over Gem-HCl, Irinotecan, and 5-FU, respectively. On the other hand, XYZ-I-71 exhibited a 2-fold increased cytotoxic effect over Gem-HCl but a comparable cytotoxic effect to 5-FU and Irinotecan in MiaPaCa-2 cells. A similar trend of higher XYZ-I-73 inhibition was observed in PANC-1 and BxPC-3 cultures. XYZ-I-73 showed a remarkably higher cytotoxic effect in PANC-1 and BxPC-3 as compared with GemHCl, 5-FU, and Irinotecan. Overall, XYZ-I-73 showed superior cytotoxicity against MiaPaCa-2, PANC-1, and BxPC-3 cells than GemHCl, 5-FU, Irinotecan, and XYZ-I-71-treated cancer cells.

This trend was expected and explains the more prominent role of higher anticancer activity of XYZ-I-73 compared to XYZ-I-71. Since XYZ-73 and XYZ-71 were synthesized using lauroyl and octanoyl acyl chains, the increased lipophilicity resulting from the conjugation could be the reason for the enhanced cellular uptake resulting in higher cytotoxic effects. Lollo et al. reported similar results when modified 5-FU with C12 chain (5-FU-C12) was used to treat HCT-116, and 9 L cell lines demonstrated prolonged duration of action (presence detected in plasma after 3 h) and higher cytotoxic activity than 5-FU(Lollo et al., 2019). In the same study, they reported that the lipid nano-capsules of 5-FU-C12 were ten times more effective than the 5-FU-C12 (Lollo et al., 2019). So, in the future, we expect a further increase in efficacy if XYZ-I-73 and XYZ-I-71 are formulated into suitable nano-drug delivery systems.

Furthermore, the selectivity of the analogs (XYZ-I-71 and XYZ -I-73) may have been enhanced by introducing a 5-membered ring with an electron-withdrawing carbonyl. Studies have shown that conjugation with a tetrahydrofuran ring makes the compound more polar and reactive than 5-FU (the parent compound), which enhances target cells’ selectivity because of its low frontier orbital energy gap and high dipole moment (Prasad et al., 2010).

Cell migration is essential for disease processes such as cancer metastasis and inflammation(Justus et al., 2014). In this study, cell migration studies were conducted at different concentrations (1 μM, 2 μM, 5 μM, and 10 μM) using novel analogs and standardized drugs (Figs. 2–5). Findings of the migration study for the 48 h revealed that XYZ-I-73 and XYZ-I-71 significantly inhibited the migration or movement of the cells towards the wound (inhibition zone) more than 5-FU and Gem-HCl. The migration of more cells toward the wound was observed in the plates treated with 5-FU. There was a significant increase in the inhibition of cell movement as the concentration of drugs increased.

When cells are subjected to various stimuli, such as DNA damage, food deprivation, viral infection, or oncogene activation, the tumor suppressor protein p53 is activated. The p53 is a tumor suppression protein, and higher expression levels of this protein indicate cytotoxicity (Lopez et al., 2015). The XYZ-I-73 showed an increasing expression of p53 as the concentration of the drug treatment increased (Fig. 6a). The slow conversion of the compound from the analog to 5-FU was able to protect the 5-FU inactivation as well as increasing the ability to suppress the growth of cancer cells. Similarly, the same trend was observed in the case of BAX protein. Overexpression of the proapoptotic gene BAX has been reported to cause apoptosis in pancreatic cancer cells. BAX activation causes the permeabilization of the mitochondrial membrane, which results in the release of the apoptotic component Cytochrome c and the death of the cancer cells (Liu et al., 2016). A significant increase in the BAX expression was observed in samples treated with IC_50_ concentrations of XYZ-I- 73 compared to 5-FU and other treatments (Fig. 6c).

PARP (poly-ADP ribose polymerase) is an important DNA repair protein. PARP inhibitors work by blocking the action of PARP when DNA damage occurs, thereby preventing repair. PARP inhibition is an indicator of cytotoxicity as its cleavage by caspases is characteristic of apoptosis (Morales et al., 2014). There was downregulation of the expression of the PARP protein in the cells treated with the IC_50_ concentrations of analogs compared to the cell samples treated with the IC_50_/2 concentrations. In contrast, when compared with 5-FU and GemHCl treated samples, the analogs had higher expressions of the PARP protein (Fig. 6b). Moreover, the results from the PARP absorbance study suggested that the XYZ-I-73 could inhibit the PARP protein by time-dependent approach (Fig. 6e). We theorize that, as we had earlier mentioned, the analogs are double prodrugs when compared to 5-FU. This could explain why samples treated with analogs showed higher PARP expression than 5-FU since they must cleave to 5-FU before they get activated and show their anticancer activity. Human liver microsomes were used to evaluate the *in-vitro* metabolic stability of XYZ-I-71 and XYZ-I-73. We found that XYZ-I-71 and XYZ-I-73 showed good stability in human liver microsomes, with more than 80 % remaining intact after 2 h, suggesting an improved *in-vitro* metabolic stability over 5-FU.

**Fig. 8.**
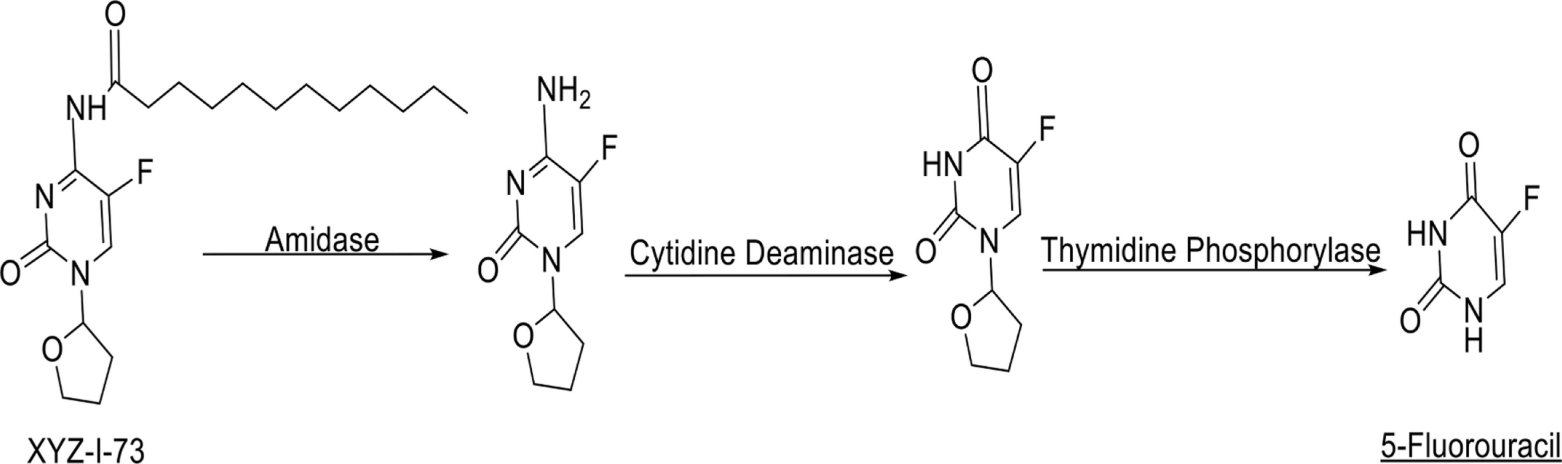
Proposed mechanism of activation of XYZ-I-73.

We attempt to propose a possible mechanism of activation (Fig. 8) for our novel pyrimidine analogs based on the literature on the activity of similar pyrimidine nucleosides in pancreatic cancer treatment (Walko and Lindley, 2005). We hypothesize that amidases hydrolyze the analog after administration to give a 5-fluorocytidine derivative. Cytidine deaminase, an enzyme found in most pancreatic cells, subsequently deaminates the 5-fluorocytidine derivative to form a 5-fluorouridine derivative. The enzyme thymidine phosphorylase, expressed in many tumor cells, then hydrolyzes the 5-fluorouridine derivative to the active drug 5-FU. This mechanism could be one of the several pathways and would have to be confirmed in subsequent studies.

Even though XYZ-I-71 showed a slightly higher cytotoxic activity over Gem-HCl, we suggest that XYZ-I-73 has greater promise as an anticancer agent for pancreatic cancer. XYZ-I-73 had a consistently higher cytotoxic effect over the standard drugs and most significantly inhibited the migration of the cells. The higher cytotoxic effects of XYZ-I-73 could result from the conjugation of the 5-fluorocytosine ring to the lauroyl hydrocarbon chain and the tetrahydrofuran ring. The conjugation may have imparted some degree of lipophilicity and target cell selectivity to XYZ-I-73. This may have facilitated its delivery to the cancer cells, a higher amount of the compounds penetrating the cell, and most likely allowed for a prolonged duration of action and ultimately improved the therapeutic efficacy of the analog.

## 5. Conclusion

In this study, we report on the design, synthesis, and biological evaluation of XYZ-I-71 and XYZ-I-73 nucleoside analogs. The XYZ-I-73 analog demonstrates a remarkable anticancer activity against pancreatic cancer cells compared with XYZ-I-71, 5-FU, Irinotecan, and GemHCl. We plan to conduct further studies on XYZ-I-73 to evaluate its toxicity, determine pharmacokinetic and biodistribution profiles, and investigate its efficacy in pancreatic PDX mouse models.

## Supplementary Material

Supplementary Data

## Figures and Tables

**Table 1:** Micro-elemental analysis for compound XYZ-I-71.

Element	Theory	Found
C	59.06	58.95
H	7.43	7.34
N	12.91	12.82

**Table 2:** Micro-elemental analysis for compound XYZ-I-73.

Element	Theory	Found
C	62.97	62.77
H	8.46	8.51
N	11.01	10.83

**Table 3:** Comparison of IC_50_ of XYZ-1–71, XYZ-I-73, 5-FU, GemHCl, and Irinotecan.

Compound	MiaPaCa-2		PANC-1		BxPC-3	
	IC_50_ (μM)	p-value	IC_50_ (μM)	p-value	IC_50_ (μM)	p-value
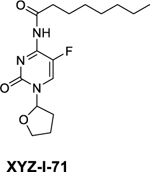	12.3 ± 1.7	12.3 ± 1.7_(XYZ-I-71)_ vs 3.6 ± 0.4 _(XYZ-I 73)_***	8.7 ± 0.9	8.7 ± 0.9 _(XYZ-I-71)_ vs 3.9 ± 0.5 _(XYZ-I-73)_*	7.7 ± 0.8	7.7 ± 0.8 _(XYZ-I-71)_ vs $5.9\pm 0.7_{\left(\text{XYZ}-1-73\right)}^{\text{ns}}$
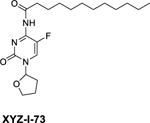	3.6 ± 0.4	3.6 ± 0.4 _(XYZ-I 73)_ *** vs 12.3 ± 1.7 _(XYZ-I-71)_	3.9 ± 0.5	3.9 ± 0.5 _(XYZ-I-73)_* vs 8.7 ± 0.9 _(XYZ-I-71)_	5.9 ± 0.7	5.9 ± 0.7 _(XYZ-I-73)_ vs $7.7\pm 0.8_{\left(\text{XYZ}-\text{I}-71\right)}^{\text{ns}}$
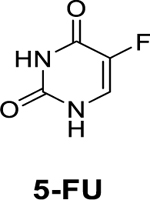	13.2 ± 1.1	12.3 ± 1.7_(XYZ-I-71)_ vs $13.2\pm 1.1_{\left(5-\text{FU}\right)}^{\text{ns}}$ 3.6 ± 0.4_(XYZ-I-73)_ *** vs 13.2 ± 1.1 _(5-FU)_	20.4 ± 1.2	8.7 ± 0.9 _(XYZ-I-71)_*** vs 20.4 ± 1.2 _(5-FU)_ 3.9 ± 0.5 _(XYZ-I-73)_*** VS 20.4 ± 0.5 _(5-FU)_	14.0 ± 1.1	7.7 ± 0.8 (XYZ-I-71)*** VS 14.0 ± 1.1 _(5-FU)_ 5.9 ± 0.7 _(XYZ-I-73)_*** VS 14.0 ± 1.1 _(5-FU)_
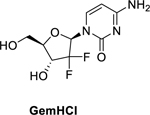	24.2 ± 1.3	12.3 ± 1.7(XYZ-I-71)*** vs 24.2 ± 1.7 (GemHCl) 3.6 ± 0.4 (XYZ-I 73)*** vs 24.2 ± 1.7 (GemHCl)	10.1 ± 0.9	8.7 ± 0.9 (XYZ-I-71) ^ns^ vs 10.1 ± 0.9 (GemHCl) 3.9 ± 0.5 (XYZ-I-73)** VS 10.1 ± 0.9 (GemHCl)	11.0 ± 0.9	7.7 ± 0.8 (XYZ-I-71)* VS 11.0 ± 0.9 _(GemHCl)_ 5.9 ± 0.7 _(XYZ-I-73)_** VS 11.0 ± 0.9 _(GemHCl)_
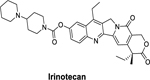	10.1 ± 1.5	12.3 ± 1.7_(XYZ-I-71)_ vs 10.1 ± 1.5 _(Iri)_^ns^ 3.6 ± 0.4_(XYZ-I-71)_*** vs 10.1 ± 1.5 _(Iri)_	11.6 ± 1.1	8.7 ± 0.9 _(XYZ-I-71)_^ns^ vs 11.6 ± 1.1 _(Iri)_ 3.9 ± 0.5 _(XYZ-I-73)_*** VS 11.6 ± 1.1 (Iri)	9.5 ± 1.0	$7.7\pm 0.8_{\left(\text{XYZ}-1-71\right)}^{\text{ns}}$ VS 9.5 ± 1.0 _(Iri)_ 5.9 ± 0.7 (XYZ-I-73)* VS 9.5 ± 1.0 _(Iri)_

Data represent ± SEM, *n* = 3

*** (*p* = 0.001

** *p* = 0.01

* *p* = 0.05 ns = not significant) Iri=Irinotecan.

**Table 4:** Quantification of XYZ-I-71, XYZ-I-73, GemHCl, and 5-FU treated cells that migrated toward the.

Number of MiaPaCa-2 cells migrated					

Concentration (μM)	Control	GemHCl	5-FU	XYZ-I-71	XYZ-I-73
1	1812 ± 6.2	878 ± 2.1	790 ± 4.3	485 ± 3.5	376 ± 2.9
2	1812 ± 6.2	786 ± 2.9	765 ± 3.4	460 ± 4.5	302 ± 3.0
5	1812 ± 6.2	762 ± 3.1	710 ± 3.2	315 ± 2.1	168 ± 2.9
10	1812 ± 6.2	682 ± 3.0	587 ± 3.1	198 ± 2.0	179 ± 1.6

wound.

Data represent ± SEM, *n* = 3.

## Data Availability

The datasets generated and analyzed during the current study are not publicly available. They are however available upon reasonable request from the corresponding author. Data will be made available on request.
