# Supplementary material for: Development of novel pyrimidine nucleoside analogs as potential anticancer agents: Synthesis, characterization, and In-vitro evaluation against pancreatic cancer
Source: Eur J Pharm Sci. Author manuscript; Available in PMC 2024 Jul 8. (PMC11229414; doi:10.1016/j.ejps.2024.106754)
Supplement: Supplementary Data [file NIHMS1985074-supplement-Supplementary_Data.pdf]

Supplementary Fig. S1

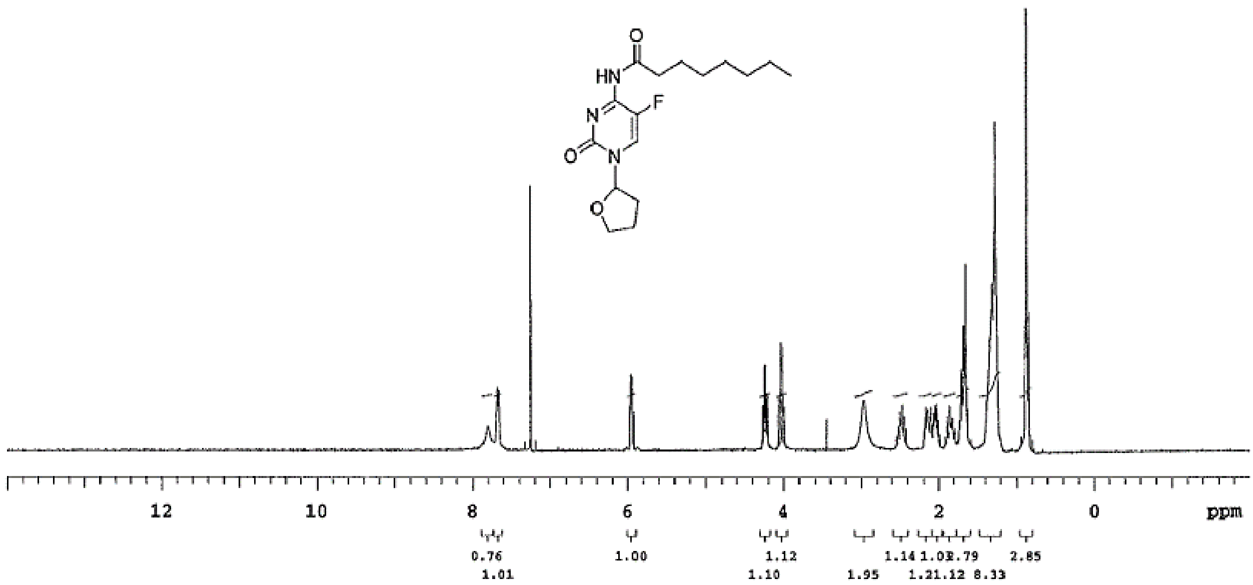

^{1}H NMR spectra for XYZ-I-71

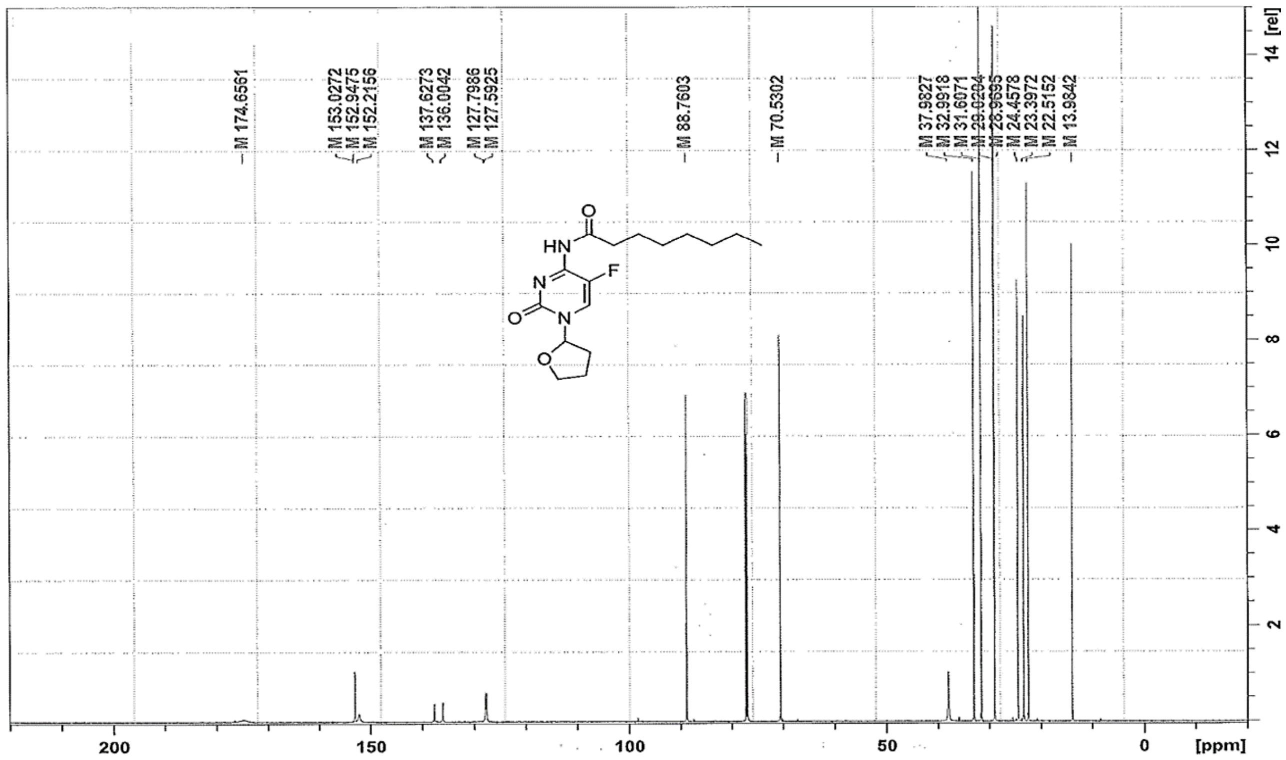

C-13 NMR spectra for XYZ-I-71

Supplementary Fig. S2

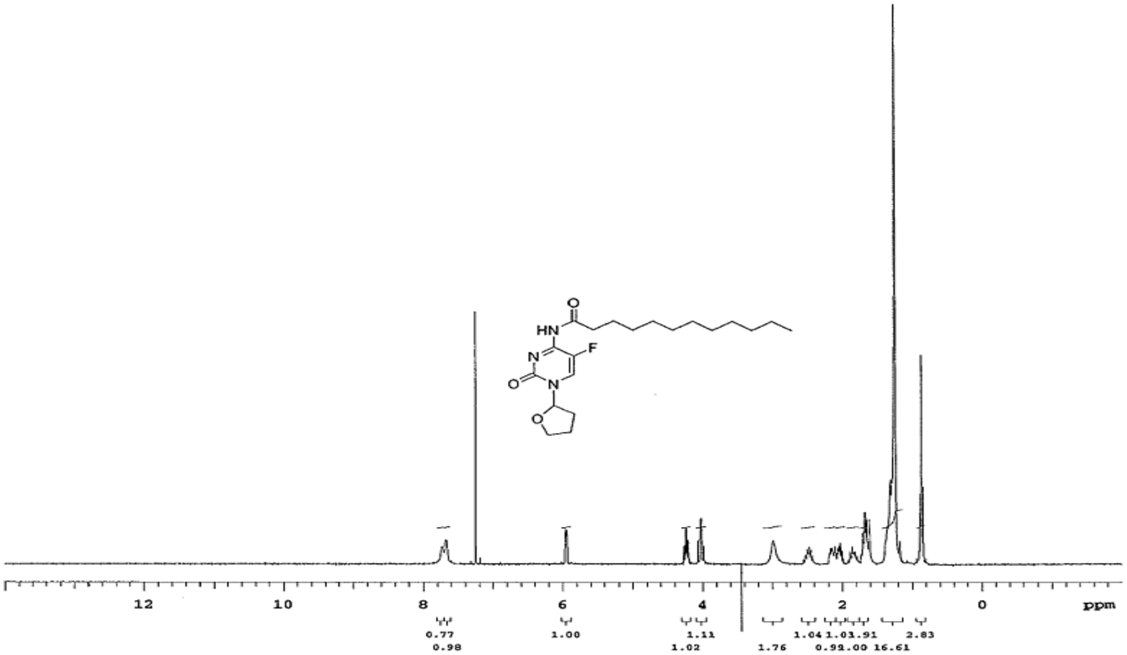

^{1}H NMR spectra for XYZ-I-73

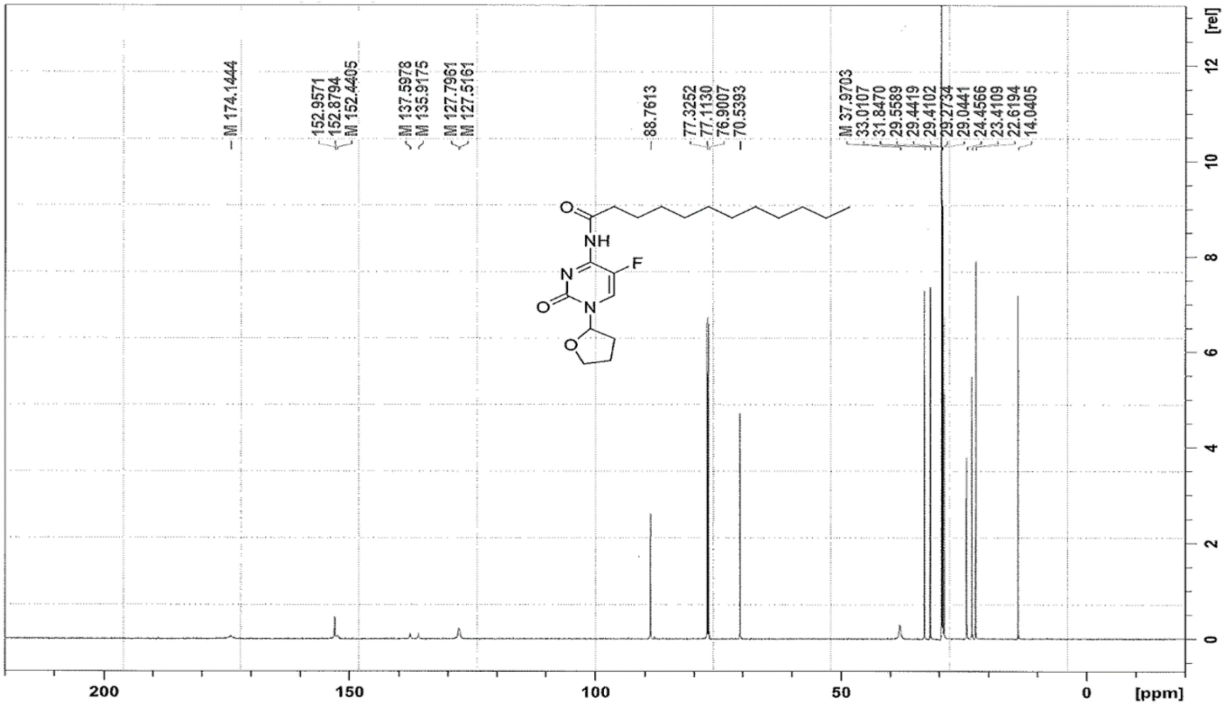

C-13 NMR spectra for XYZ-I-73

Supplementary Fig. S3

Atlantic Microlab, Inc.

Sample No. XYZ-I-71

6180 Atlantic Blvd. Suite M
Norcross, GA 30071
www.atlanticmicrolab.com

Company/School: Florida A&M Univ
Dept.: College of Pharmacy
Address:
City, State, Zip:

Professor/Supervisor: Dr Agyare
PO# / CC#: FAM01-0000186402

Name: Xue You Zhu   Date: 06/22/2020
Phone:

Element	Theory	Found	
C	59.06	58.95	
H	7.43	7.34	
N	12.91	12.82	

Single ☐ Duplicate ☐
Elements Present: C16H24FN3O3
Analyze for: C H N
Hygroscopic ☐ Explosive ☐
M.P. ______ B.P. ______
To be dried: Yes ☒ No ☐
Temp. rt Vac. yes Time 24 h
Rush Service ☐ Rush service guarantees analyses will be completed and results available by 5 PM EST on the day the sample is received by 11 AM.
Include Email Address or FAX # Below
xue.zhu@famu.edu

Date Received JUN 25 2020   Date Completed JUN 26 2020
Remarks:

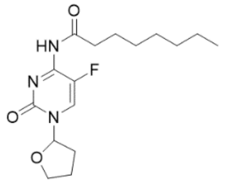

Chemical Formula: $C_{16}H_{24}FN_3O_3$
Molecular Weight: 325.38
Elemental Analysis: C, 59.06; H, 7.43; N, 12.91
Log P: 2.27

Micro-elemental analysis of XYZ-I-71showing the percent by mass of the elements carbon, hydrogen, and nitrogen in the structure of XYZ-I-71compared to theoretical estimates.

Supplementary Fig. S4

Atlantic Microlab, Inc.

Sample No. XYZ-I-73

6180 Atlantic Blvd. Suite M
Norcross, GA 30071
www.atlanticmicrolab.com

Company/School Florida A&M Univ
Dept. College of Pharmacy
Address
City, State, Zip

Professor/Supervisor: Dr Agyare
PO# / CC# FAM01-0000186402

Name Xue You Zhu Date 06/22/2020
Phone

Element	Theory	Found	
C	62.97	62.77	
H	8.46	8.51	
N	11.01	10.83	

Single ☐ Duplicate ☐
Elements Present: C20H32FN3O3
Analyze for: C H N
Hygroscopic ☐ Explosive ☐
M.P. B.P.
To be dried: Yes ☒ No ☐
Temp. rt Vac. yes Time 24 h
Rush Service ☐ Rush service guarantees analyses will be completed and results available by 5 PM EST on the day the sample is received by 11 AM.
Include Email Address or FAX # Below
xue.zhu@famu.edu

Date Received JUN 25 2020 Date Completed JUN 26 20[illegible]
Remarks:

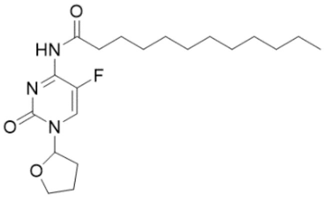

Chemical Formula: $C_{20}H_{32}FN_3O_3$
Molecular Weight: 381.49
Elemental Analysis: C, 62.97; H, 8.46; N, 11.01;
Log P: 3.94

Micro-elemental analysis of XYZ-I-73 showing the percent by mass of the elements carbon, hydrogen, and nitrogen in the structure of XYZ-I-73 compared to theoretical estimates.

Supplementary Fig. S5

Additional Info : Peak(s) manually integrated

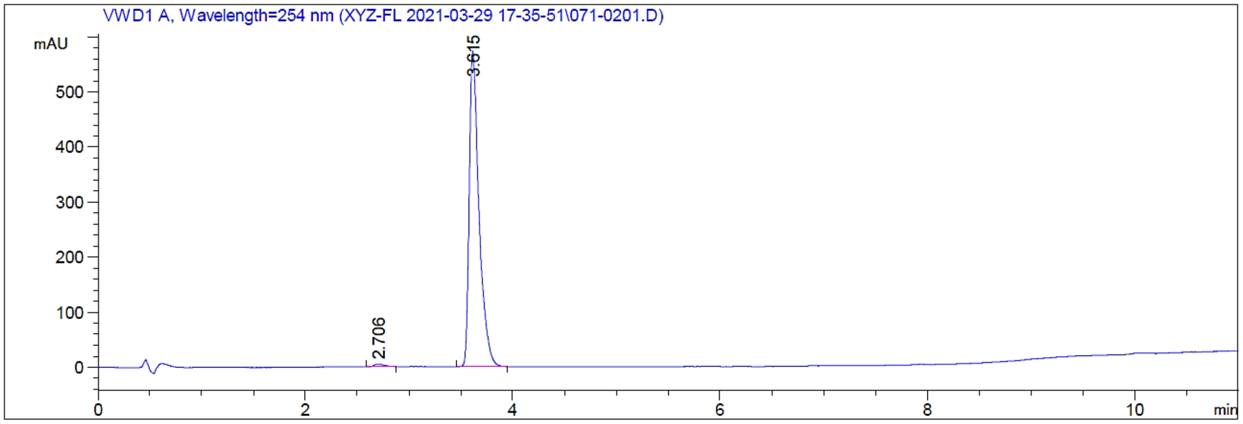

===

Area Percent Report

===

Sorted By : Signal
Multiplier : 1.0000
Dilution : 1.0000
Use Multiplier & Dilution Factor with ISTDs

Signal 1: VWD1 A, Wavelength=254 nm

Peak #	RetTime [min]	Type	Width [min]	Area mAU *s	Height [mAU]	Area %
1	2.706	BV	0.0778	32.30576	5.01077	0.8473
2	3.615	VV	0.0976	3780.58179	574.17224	99.1527

Totals : 3812.88755 579.18301

HPLC analysis of XYZ-I-71 showing a sharp peak at retention time of 3.615, performed by an independent laboratory.

Supplementary Fig. S6

Additional Info : Peak(s) manually integrated

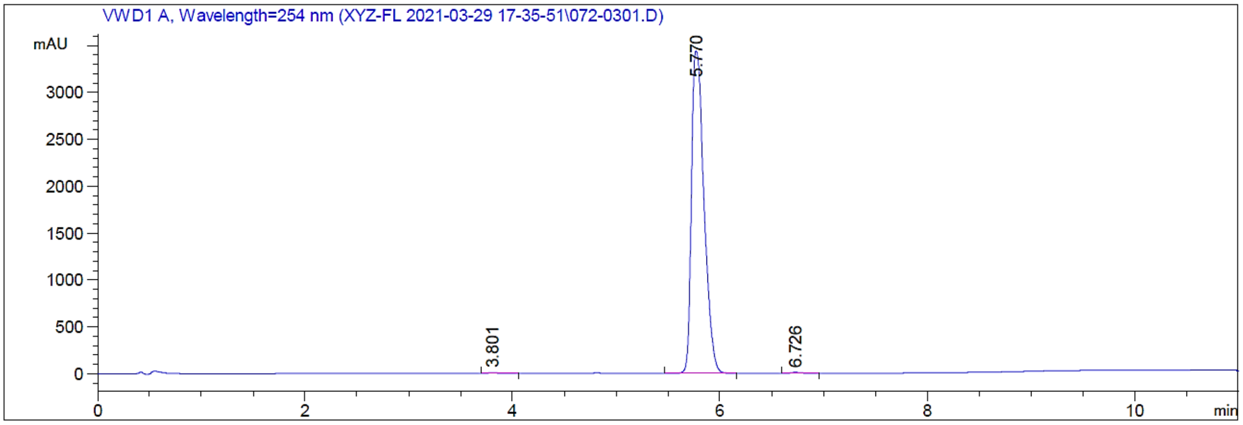

===

Area Percent Report

===

Sorted By : Signal
Multiplier : 1.0000
Dilution : 1.0000
Use Multiplier & Dilution Factor with ISTDs

Signal 1: VWD1 A, Wavelength=254 nm

Peak #	RetTime [min]	Type	Width [min]	Area mAU *s	Height [mAU]	Area %
1	3.801	VV	0.0939	61.62813	9.54056	0.2128
2	5.770	BV	0.1120	2.88379e4	3439.35571	99.5618
3	6.726	BB	0.0976	65.31019	9.96237	0.2255
Totals :				2.89649e4	3458.85865	

HPLC analysis of XYZ-I-73 showed a sharp peak at a retention time of 5.770, performed by an independent laboratory.

Supplementary Fig. S7

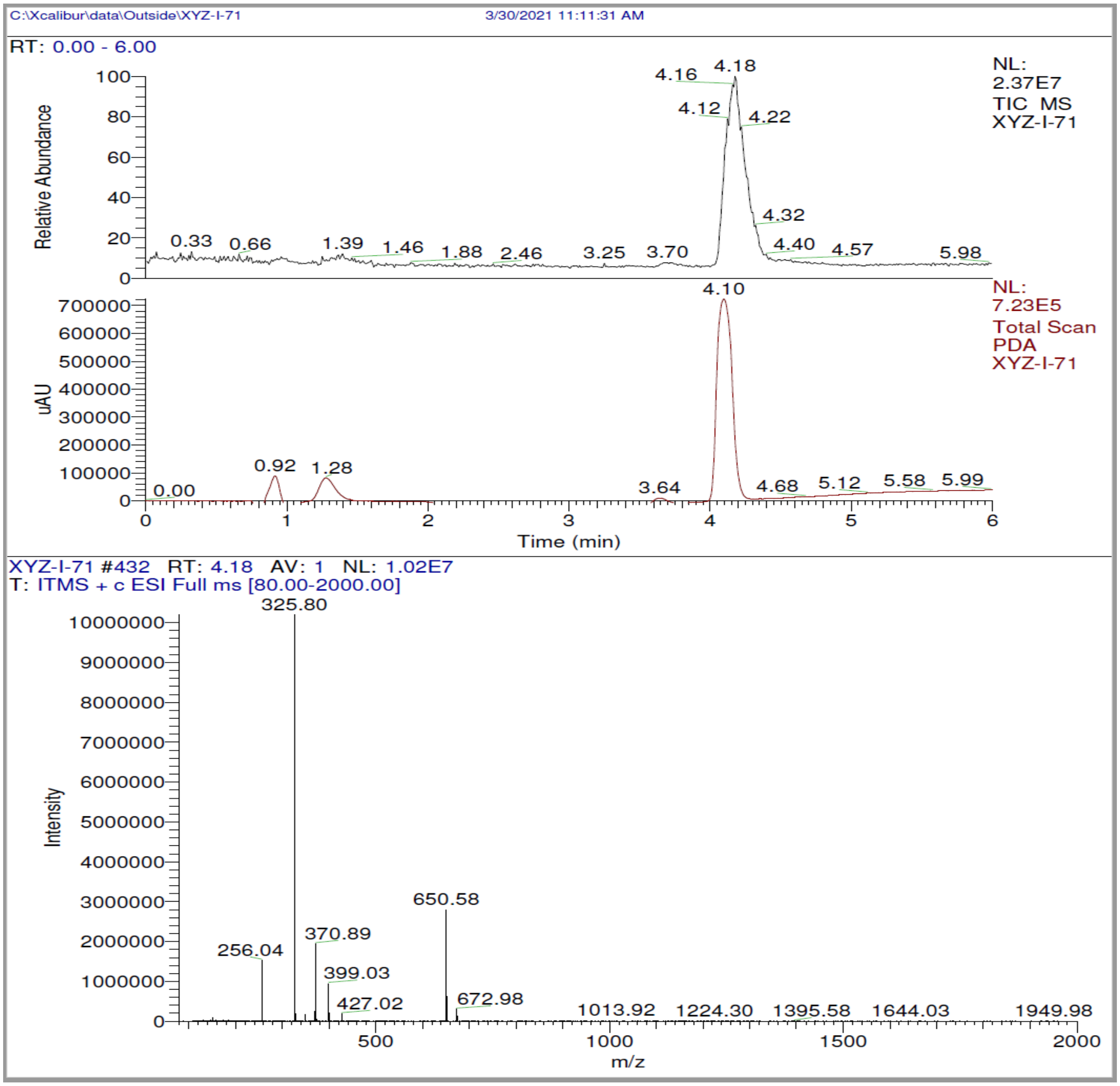

LCMS analysis of XYZ-I-71

Supplementary Fig. S8

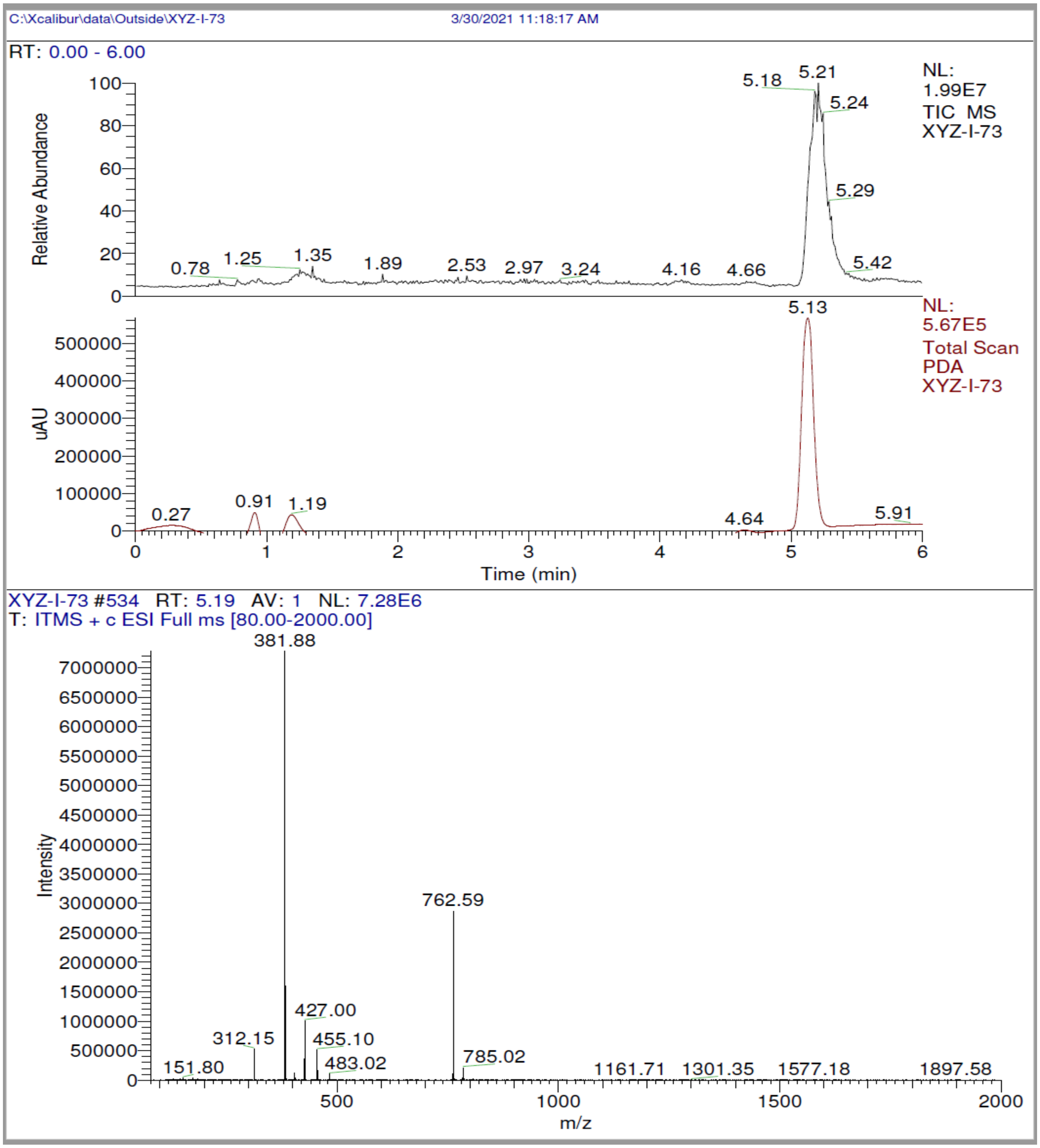

LCMS analysis of XYZ-I-73

Supplementary Fig. S9

A) B-actin

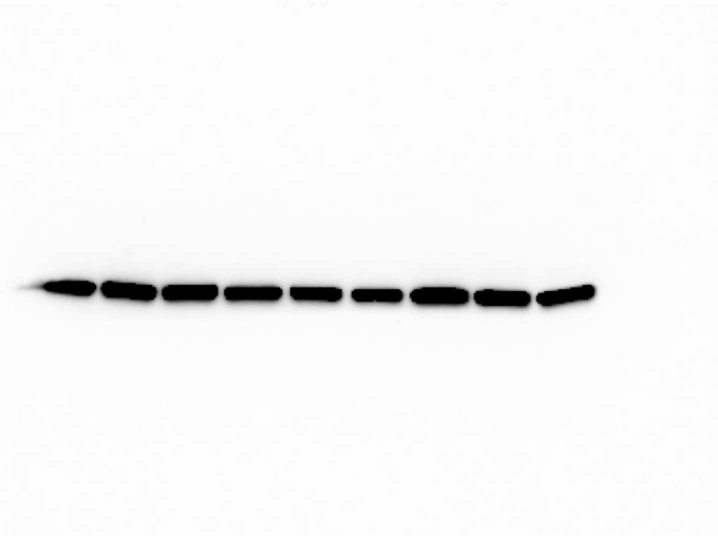

B) BAX

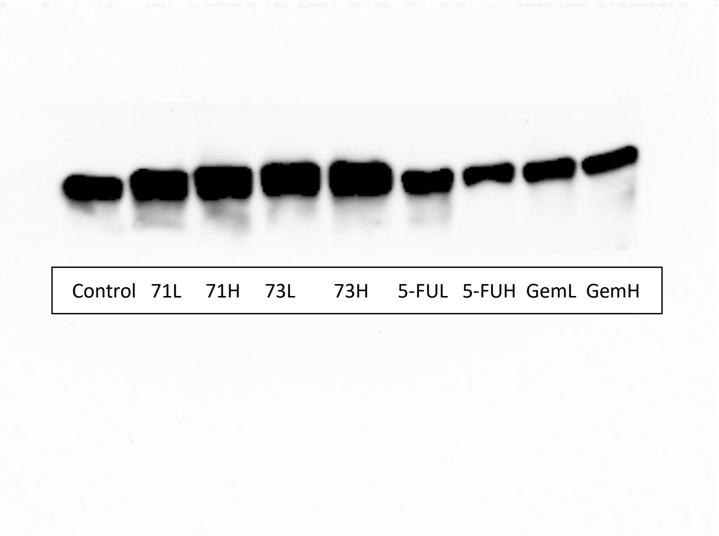

C) p53

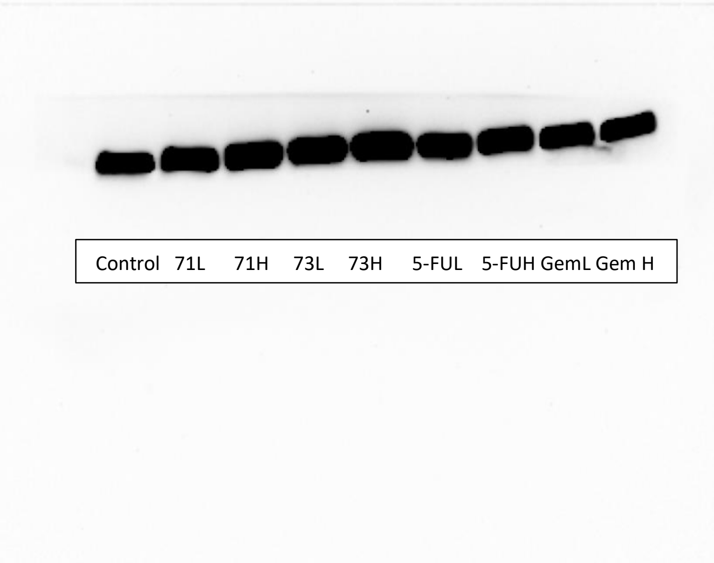

D) PARP

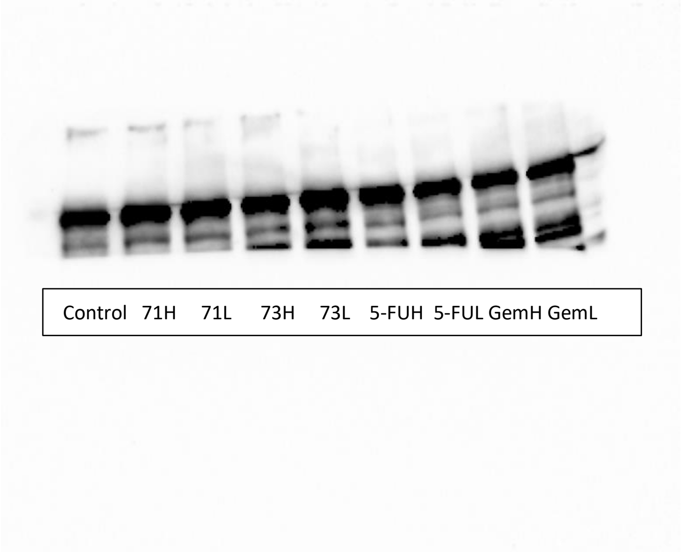

Full-length blots of B-actin, BAX, p53, and PARP expression after treatment of MiaPaca-2 cells with IC_{50} and $IC_{50}/2$ concentrations of XYZ-I-71, XYZ-1-73, 5-FU, and GemHCl. $IC_{50}/2$ and IC_{50} concentrations for respective drugs are indicated as L & H, respectively.
